# Almost Surely Exponential Convergence Analysis of Time Delayed Uncertain Cellular Neural Networks Driven by Liu Process via Lyapunov–Krasovskii Functional Approach

**DOI:** 10.3390/e25111482

**Published:** 2023-10-26

**Authors:** Chengqiang Wang, Zhifu Jia, Yulin Zhang, Xiangqing Zhao

**Affiliations:** 1 School of Mathematics, Suqian University, Suqian 223800, China; 2 School of Mathematics, Chengdu Normal University, Chengdu 611130, China

**Keywords:** cellular neural networks, Liu process, time delays, equilibrium states, convergence analysis, uncertainty theory, Lyapunov–Krasovskii functionals

## Abstract

As with probability theory, uncertainty theory has been developed, in recent years, to portray indeterminacy phenomena in various application scenarios. We are concerned, in this paper, with the convergence property of state trajectories to equilibrium states (or fixed points) of time delayed uncertain cellular neural networks driven by the Liu process. By applying the classical Banach’s fixed-point theorem, we prove, under certain conditions, that the delayed uncertain cellular neural networks, concerned in this paper, have unique equilibrium states (or fixed points). By carefully designing a certain Lyapunov–Krasovskii functional, we provide a convergence criterion, for state trajectories of our concerned uncertain cellular neural networks, based on our developed Lyapunov–Krasovskii functional. We demonstrate under our proposed convergence criterion that the existing equilibrium states (or fixed points) are exponentially stable almost surely, or equivalently that state trajectories converge exponentially to equilibrium states (or fixed points) almost surely. We also provide an example to illustrate graphically and numerically that our theoretical results are all valid. There seem to be rare results concerning the stability of equilibrium states (or fixed points) of neural networks driven by uncertain processes, and our study in this paper would provide some new research clues in this direction. The conservatism of the main criterion obtained in this paper is reduced by introducing quite general positive definite matrices in our designed Lyapunov–Krasovskii functional.

## 1. Introduction

By simulating the function of the biological brain, neural networks (NNs), the totality of mathematics-based computational models, deliver outstanding performance in recognition and/or classification of patterns, signal processing, engineering optimization, associative memory and so forth. Therefore, various types of NNs have been created for specific purposes; see [1,2,3,4,5,6,7,8,9,10,11] and the vast references cited therein. For example, in the 1980s, Chua and Yang invented a class of NNs, called cellular NNs (CNNs); see [1,2] for the details. Different from the classical Hopfield NNs (HNNs), the (dynamical) behavior of nodes (cells) of CNNs can merely be influenced by their nearly neighboring nodes (cells); see [1,2,12,13,14]. In the last three decades, CNNs have been extensively applied in diverse areas such as image processing, associative memory and parallel computing. As a consequence, CNNs have been extensively and intensively investigated, among scientific and engineering communities, in recent years from the point of view of mathematics (dynamical system theory and/or mathematical control theory, say); see References [3,6,10,11,12,13,14], for example.

The convergence property of state trajectories to equilibrium states (or fixed points) of NNs is usually referenced as the stability of equilibrium states (or fixed points), and is termed occasionally as the stability of the NNs. Convergence is one of the key properties to guarantee the NNs be successfully applied in the engineering community. And therefore, CNNs have been widely investigated for the existence and the stability of equilibrium states (or fixed points); see the pioneering works [1,12,13] and the references mentioned therein. For example, Chua and Yang [1] briefly discussed the importance of studying the existence and stability of equilibrium states (or fixed points) of CNNs via using dynamical system theory, and obtained some interesting existence and convergence criteria.

As is well known to all, probability theory has been widely used to model the indeterminacy phenomena brought about by randomness of the environment; see References [4,6,11,15]. In recent years, Liu has developed, based on the understanding an individual’s belief degree, uncertainty theory to portray the indeterminacy in individual’s subjective cognition; see Reference [16] (see also [17,18] and the vast references therein). Since its birth date, uncertainty theory has attracted extensive attentions in both the research and application communities. Up to now, uncertainty theory nearly has all theoretical results parallel to probability theory; see References [16,17,18,19,20,21,22,23,24,25,26,27,28,29]. In particular, the theory of uncertain processes and the uncertain calculus have been already developed well; see Reference [16] (see also [17,18,28], say). The well-established uncertain calculus has paved the area of studying behaviors of dynamical systems subject to uncertain perturbations (for example, the concerned dynamics is driven by the so-called canonical Liu process). In this paper, we shall consider a class of NNs whose dynamics is driven by the canonical Liu process.

It would certainly cost time for nodes (cells) themselves to process information and in the procedure of transmission of information between every pair of nodes (cells), therefore, time delay exists unavoidably in real world NNs; see [3,4,5,6,9,10,12,14,15,23,30,31], among the vast existing references. Generally speaking, time delay in NNs would certainly bring about more challenges in proving the convergence of state trajectories of NNs.

By reviewing the aforementioned references, we are inspired to study time-delayed uncertain CNNs (DUCNNs) for the convergence of their state trajectories. One of the main aims in this respect is to provide a criterion ensuring the existence of equilibrium states (or fixed points); and another main aim is to put forward a criterion to guarantee that state trajectories of the concerned DUCNNs converge to equilibrium states (or fixed points). In this direction, some interesting results have already been published in the literature. As alluded to previously, the existence and stability of equilibrium states (or fixed points) of deterministic CNNs (whose dynamics is not influenced by stochastic or uncertain environment) were discussed in References [1,12,13]. (Almost) periodic state trajectories of NNs play similar roles as equilibrium states (or fixed points). Kong, Zhu et al. [5] studied a class of discontinuous bidirectional associative memory NNs (briefly referenced as BAMNNs, and can be viewed as a specific class of CNNs) with hybrid time-varying delays and *D* operator, and obtained a criterion ensuring the existence of almost periodic state trajectories of their concerned BAMNNs, and provided another criterion guaranteeing the stability of the almost periodic state trajectories. We shall discuss briefly (almost) periodic state trajectories of NNs in Section 5 again. When the dynamics of CNNs is influenced by some stochastic environment, the problem concerning the almost surely exponential stability almost stochastic was investigated in [14,15,30,31,32,33,34] and some related references therein. For example, Cong [33] obtained an interesting robust almost sure stability result for continuous-time linear systems subject to exogenous disturbance.

As with stochastic dynamical systems driven by Brownian motions (Lévy processes, semimartingales with/without jumps, and so on), the problem concerning the existence of state trajectories for uncertain dynamical systems driven by canonical processes has aroused extensive research interest in recent years. In this respect, a large number of meaningful results were published; see References [16,17,18,21,29,35], for example. Chen and Liu [18] considered general uncertain differential equations and obtained some important (unique) existence results. Shu and Li [35] studied a class of switched nonlinear uncertain systems and proved, via the Contraction Mapping Principle, the existence and uniqueness of state trajectories for their concerned systems. The results concerning the convergence property of state trajectories or the stability of equilibrium states (or fixed points) can be seen in References [21,22,23,24,25,26,27,28,29]. Jia and Liu [29] studied, besides the (unique) solvability, the convergence property of age-dependent uncertain population equations subject to stochastic perturbations. Lu and Zhu [23] investigated a class of uncertain dynamical systems, and came up with several criteria ensuring the convergence of state trajectories of their concerned time delayed uncertain dynamical systems in the sense of moments. Jia and Li [21] obtained a criterion to ensure almost sure exponential stability of uncertain HNNs (UHNNs) under stochastic perturbations. For (deterministic, stochastic, uncertain) dynamical systems, the continuous dependence of state trajectories on initial states is also very important. In the context of uncertain dynamical systems, the continuous dependence of state trajectories on initial states is frequently referenced as the stability (of the concerned dynamical systems). Yao, Gao et al. [36] obtained some general stability (continuous dependence) theorems of uncertain differential equations. In References [19,20], some interesting stability (continuous dependence) results in the mean sense for uncertain differential equation were obtained. Some other interesting stability (continuous dependence) results can also be seen in References [22,28] and the references therein.

Our principal contributions in this paper are as follows.

- We investigate in-depth in this paper a class of DUCNNs driven by a canonical Liu process for the stability of their equilibrium states (or fixed points). As alluded to previously, the dynamics of NNs is inevitably influenced by a random (stochastic) environment. And analogously, humans’ subjective cognition based on intuition or inspection (in terms of belief degree) may have some influence on the structure of NNs when the workers are designing the NNs, and therefore have a certain influence on the dynamics of the constructed NNs. Therefore, the research results concerning uncertain NNs may be more suitable and reliable for the decision makers. Uncertainty theory laid the foundation (the notion of belief degree on measurable spaces) of the mathematical theory that are capable of portraying quantitatively humans’ subjective cognition. Therefore, it is of great importance to study uncertain NNs for the large time behavior of their state trajectories. By reviewing the existing references, we conclude that our research results in this paper seem to be new. For example, in comparison with Reference [23], in which stability criteria were provided in terms of moments, our aim in this paper is to provide a stability criterion in the sample sense for DUCNNs. For another example, in contrast with Reference [21], our concerned model DUCNNs include discrete time and finitely distributed time delay.
- Via applying the classical Contraction Mapping Principle, we establish a criterion (see Theorem 1 in Section 3), and prove that this criterion can guarantee that our concerned model DUCNNs have unique equilibrium states (or fixed points).
- We design meticulously a class of Lyapunov–Krasovskii functionals, which take into account the after-effect (or time delay) in our concerned model DUCNNs, analyze in detail our concerned model DUCNNs with these coined Lyapunov–Krasovskii functionals as the key tools, and establish a criterion to ensure that equilibrium states (or fixed points) of our concerned model DUCNNs be almost surely exponentially stable; see Theorem 2 in Section 3. We also come up with a specific example DUCNN to validate our theoretical results; see Section 4.

**Notational Conventions.**  *We write R for the totality of real numbers, and $R_{+}$ for the interval [0,+∞) of non-negative real numbers. We write N for a positive integer throughout this paper. We denote by $R^{N}$ the N-dimensional Euclidean space, and by $R^{N\times N}$ the algebra of N-th order real square matrices. Following the common convention, we designate by (R,L,dt) the usual Lebesgue measure space. We denote by (Γ,L,L,M) (or (Γ,L,L,dM)) a complete filtered uncertainty space (whose definition would be explained in detail in Section 2; see Definition 1), in which, the filtration $L=\{L_{t};\;t\in R_{+}\}$ is assumed to satisfy the usual conditions; that is, the σ-algebra $L_{0}$ contains all M-null sets in the σ-algebra L, and the filtration L is right-continuous in the sense that*

(1) $$\begin{array}{cccc} \; & \underset{s>t}{⋂}L_{s}=L_{t}, & \; & t\in R_{+}. \end{array}$$

*“L almost surely” is abbreviated as M-a.s. Let X be an arbitrarily given uncertain variable on Γ, denote by EX (see Definition 5) the expected value of X, and by ${𝘍}_{\xi }\left(x\right)$ (see Definition 4) the uncertainty distribution ${𝘍}_{\xi }\left(x\right)$ of ξ. (Γ×R,L⊗L,dM×dt) denotes the product σ-subadditive measure space of (R,L,dt) and (Γ,L,M); $\left\{C\left(t\right);\;t\in R_{+}\right\}$, an L-adapted uncertain process, denotes a one dimensional canonical Liu process defined on the uncertainty space (Γ,L,L,M). Let A be a positive definite matrix, we write $\lambda_{\min}\left(A\right)$ and $\lambda_{\max}\left(A\right)$, respectively, for the smallest and largest eigenvalues of A. Let A be a square matrix, which we denote by trA (or tr(A), occasionally) the trace of A, and by sym(A) the symmetric matrix $A+A^{⊤}$ with $A^{⊤}$ designating the transpose of A here and hereafter. For any positive definite matrix $A\in R^{N\times N}$, we designate its Cholesky decomposition by ${⨿}_{A}^{⊤}{⨿}_{A}$ with ${⨿}_{A}$ an upper triangular matrix (${⨿}_{A}$ is actually nonsingular and unique). For any pair of symmetric matrices A and $B\in R^{N\times N}$, if A−B is positive definite, then we write A≻B. In particular, if the matrix $A\in R^{N\times N}$ is positive definite, then we write A≻0.*

The rest of this paper is organized as follows. In Section 2, we recall some preliminaries necessary for our later presentation and formulate our concerned existence and convergence problems for DUCNNs. In Section 3, we state the principal results in this paper and present their proofs in detail. In Section 4, we justify, in both numeric and visual ways, the effectiveness of our theoretical results, via bringing forward a specific example DUCNN of which state trajectories converge to the unique equilibrium state (or fixed point). In Section 5, we conclude our discussion in this paper by presenting several remarks.

## 2. Preliminaries and Formulation of the Problems

### 2.1. Some Preliminaries

Let (Γ,L) be a measurable space with Γ a nonempty set and L a σ-algebra over Γ. We equip (Γ,L) throughout this paper the filtration $L=\{L_{t};\;t\in R_{+}\}$ satisfying the usual conditions. In other words, L is a collection of sub-σ-algebra of L and satisfies (i) The σ-algebra $L_{0}$ contains all M-null sets in the σ-algebra L; and (ii) L is right-continuous in the sense of (1). Here and hereafter, we shall write (Γ,L,L) for the measurable space (Γ,L) equipped with the filtration $L=\{L_{t};\;t\in R_{+}\}$ satisfying the usual conditions.

**Definition 1.**  *Given a measurable space (Γ,L,L), equipped with a filtration $L=\{L_{t};\;t\in R_{+}\}$ that satisfies the usual conditions, and a given function M mapping L into [0,1]. The given function M is called a uncertainty measure on the filtered measurable space (Γ,L,L) provided that the following three axioms are fulfilled:*

- *(Normality). It holds always that M(Γ)=1;*
- *(Self-duality). It holds always that M(Γ\A)=1−M(A) for every event A in L;*
- *(Countable subadditivity). For every sequence ${\left\{A_{n}\right\}}_{n=1}^{\infty }$ in L, it holds always that*
$$\begin{array}{c} M\left(\overset{\infty }{\underset{n=1}{⋃}}A_{n}\right)⩽\sum_{n=1}^{\infty }M\left(A_{n}\right). \end{array}$$

*The quadruple (Γ,L,L,M), obtained by equipping the filtered measurable (Γ,L,L) with the uncertainty measure M, is called a uncertainty space.*

From now on, we abide by the convention that (Γ,L,L,M) is a complete filtered uncertainty space in which the filtration L satisfies the usual conditions.

**Definition 2.**  *The measurable function ξ:Γ→R is called a uncertain variable. In more detail, if for any Borel subset B of R, then the set*

$$\begin{array}{cc} \xi^{-1}\left(B\right)= & \left\{\gamma \in \Gamma ;\;\xi (\gamma )\in B\right\}\\ = & \left\{\gamma \in \Gamma ;\;\exists x\in B,\;such\;that\;\xi (\gamma )=x\right\} \end{array}$$

*belongs to the σ-algebra L, then ξ is said to be a uncertain variable.*

**Definition 3.**  *Let ξ be a uncertain variable on the uncertainty space (Γ,L,L,M). The following associated real-valued function*

(2) $$\begin{array}{cccc} \; & {𝘍}_{\xi }\left(x\right)=M\left(\left\{\gamma \in \Gamma ;\;\xi \left(\gamma \right)⩽x\right\}\right),\; & \; & x\in R \end{array}$$

*is called the uncertainty distribution of ξ.*

**Definition 4.**  *Let ξ be a uncertain variable on the uncertainty space (Γ,L,L,M). If the uncertainty distribution ${𝘍}_{\xi }\left(x\right)$ of ξ is exactly*

(3) $$\begin{array}{cccc} {𝘍}_{\xi }\left(x\right)= & \Phi (\frac{x-\xi_{0}}{\sigma }) & \; & \\ = & \frac{1}{1+e^{\frac{\pi (\xi_{0}-x)}{\sqrt{3}\sigma }}},\; & \; & x\in R \end{array}$$

*with $\xi_{0}$ a given constant in R and σ a given positive constant, then we call ξ a normal uncertain variable with expected value $\xi_{0}$ and variance $\sigma^{2}$. If $\xi_{0}=0$ and σ=1, we call ξ a standard normal uncertain variable, and write its uncertainty distribution as*

(4) $$\begin{array}{cccc} \; & \Phi \left(x\right)=\frac{1}{1+e^{-\frac{\pi x}{\sqrt{3}}}},\; & \; & x\in R. \end{array}$$

It is obvious that the function Φ(x) given by (4) is strictly increasing in R. We can conclude therefore that the function Φ(x) has inverse function $\Phi^{-1}\left(x\right)$. Actually, by some routine calculations, we have immediately

(5) $$\begin{array}{cccc} \; & \Phi^{-1}\left(x\right)=\frac{\sqrt{3}}{\pi }\ln\frac{x}{1-x},\; & \; & x\in (0,1). \end{array}$$

We shall call the function $\Phi^{-1}\left(x\right)$ (the inverse function of Φ(x) given by (4)), given as in (5), the inverse standard normal uncertainty distribution throughout this paper.

**Definition 5.**  *Suppose that ξ is an uncertain variable on the uncertainty space (Γ,L,L,M). If at least one of the following two integrals:*

$$\int_{0}^{+\infty }M\left(\left\{\gamma \in \Gamma ;\;\xi \left(\gamma \right)⩾x\right\}\right)dx$$

*and*

$$\int_{-\infty }^{0}M\left(\left\{\gamma \in \Gamma ;\;\xi \left(\gamma \right)⩽x\right\}\right)dx$$

*are finite, then we call*

$$\begin{array}{cc} E\xi = & \int_{0}^{+\infty }M\left(\left\{\gamma \in \Gamma ;\;\xi \left(\gamma \right)⩾x\right\}\right)dx\\ \; & -\int_{-\infty }^{0}M\left(\left\{\gamma \in \Gamma ;\;\xi \left(\gamma \right)⩽x\right\}\right)dx \end{array}$$

*the expected value of the uncertain variable ξ.*

Based on the definitions of Eξ and ${𝘍}_{\xi }\left(x\right)$, it is straightforward to verify that

(6) $$E\xi =\int_{0}^{+\infty }(1-{𝘍}_{\xi }\left(x\right))dx-\int_{-\infty }^{0}{𝘍}_{\xi }\left(x\right)dx.$$

This identity facilitates the calculations of expected values of uncertain variables. To provide some intuitions for our later theoretical development in this paper, we would like to share the next two examples on the computations of expected values of uncertain variables.

**Example 1.**  *Let ξ be a normal uncertain variable, with expected value $\xi_{0}$ and variance $\sigma^{2}$, on the uncertainty space (Γ,L,L,M). The expected value Eξ of ξ is equal to $\xi_{0}$. Indeed, based on *(6)*, we deduce from *(4)* that*

(7) $$\begin{array}{cc} E\xi = & \int_{0}^{+\infty }(1-{𝘍}_{\xi }\left(x\right))dx-\int_{-\infty }^{0}{𝘍}_{\xi }\left(x\right)dx\\ = & \int_{0}^{+\infty }(1-\Phi \left(\frac{x-\xi_{0}}{\sigma }\right))dx-\int_{-\infty }^{0}\Phi \left(\frac{x-\xi_{0}}{\sigma }\right)dx\\ = & \int_{0}^{+\infty }\frac{e^{\frac{\pi (\xi_{0}-x)}{\sqrt{3}\sigma }}}{1+e^{\frac{\pi (\xi_{0}-x)}{\sqrt{3}\sigma }}}dx-\int_{-\infty }^{0}\frac{dx}{1+e^{\frac{\pi (\xi_{0}-x)}{\sqrt{3}\sigma }}}\\ = & -\frac{\sqrt{3}\sigma }{\pi }\int_{-\infty }^{0}\frac{1}{1+e^{\frac{\pi (x-\xi_{0})}{\sqrt{3}\sigma }}}d\left(1+e^{\frac{\pi (x-\xi_{0})}{\sqrt{3}\sigma }}\right)\\ \; & -\frac{\sqrt{3}\sigma }{\pi }\int_{0}^{+\infty }\frac{1}{1+e^{\frac{\pi (\xi_{0}-x)}{\sqrt{3}\sigma }}}d\left(1+e^{\frac{\pi (\xi_{0}-x)}{\sqrt{3}\sigma }}\right)\\ = & \frac{\sqrt{3}\sigma }{\pi }(\ln\left(1+e^{\frac{\pi \xi_{0}}{\sqrt{3}\sigma }}\right)-\ln\left(1+e^{-\frac{\pi \xi_{0}}{\sqrt{3}\sigma }}\right))=\xi_{0}. \end{array}$$

**Example 2.**  *Let ξ be a normal uncertain variable, with expected value $\xi_{0}$ and variance $\sigma^{2}$, on the uncertainty space (Γ,L,L,M). Following the steps to derive *(7)* in Example 1, we have*

$$\begin{array}{cc} Ee^{\xi }= & \int_{0}^{+\infty }(1-{𝘍}_{e^{\xi }}\left(x\right))dx-\int_{-\infty }^{0}{𝘍}_{e^{\xi }}\left(x\right)dx=\int_{0}^{+\infty }(1-\Phi \left(\frac{\ln x-\xi_{0}}{\sigma }\right))dx\\ = & \int_{0}^{+\infty }\frac{e^{\frac{\pi (\xi_{0}-\ln x)}{\sqrt{3}\sigma }}}{1+e^{\frac{\pi (\xi_{0}-\ln x)}{\sqrt{3}\sigma }}}dx=e^{\xi_{0}}\int_{0}^{+\infty }\frac{1}{1+{\left(e^{-\xi_{0}}x\right)}^{\frac{\pi }{\sqrt{3}\sigma }}}d\left(e^{-\xi_{0}}x\right)\\ = & \left\{\begin{array}{ccc} \frac{\sqrt{3}\sigma e^{\xi_{0}}}{\pi }B\left(\frac{\sqrt{3}\sigma }{\pi },1-\frac{\sqrt{3}\sigma }{\pi }\right)=\sqrt{3}\sigma e^{\xi_{0}}\csc\left(\sqrt{3}\sigma \right) & \; & if\;\sigma \in (0,\frac{\pi }{\sqrt{3}}),\\ +\infty & \; & if\;\sigma \in [\frac{\pi }{\sqrt{3}},+\infty ), \end{array}\right. \end{array}$$

*where B(∗,★) is Euler’s Beta function.*

**Definition 6.**  *Let T be a nonempty subset of $R_{+}$. The function X:Γ×T→R is said to be an uncertain process provided that it is progressively measurable.*

**Definition 7.**  *Let ${\left\{C\left(t\right)\right\}}_{t\in R_{+}}$ be an uncertain process. The given process ${\left\{C\left(t\right)\right\}}_{t\in R_{+}}$ is called a canonical Liu process provided that the following three assertions hold:*

- *M({γ∈Γ;C(γ,0)≠0})=0, and*
$$M(\left\{\gamma \in \Gamma ;\;C\left(\gamma ,t\right)\;\mathrm{is}\;\mathrm{Lipschitz}\;\mathrm{continuous}\;\mathrm{in}\;R_{+}\right\})=1;$$
- *${\left\{C\left(t\right)\right\}}_{t\in R_{+}}$ has stationary and independent increments;*
- *For every $t\in R_{+}$ and every s∈(0,+∞), the increment C(t+s)−C(s) is a normal uncertain variable with expected value 0 and variance $t^{2}$.*

**Definition 8.**  *Let ${\left\{C\left(t\right)\right\}}_{t\in R_{+}}$ be the aforementioned canonical Liu process. We denote*

(8) $$\begin{array}{cccc} \; & k\left(\gamma \right)=\underset{\begin{array}{c} s,t\in R_{+},\\ s\neq t \end{array}}{\sup}\left|\frac{C(\gamma ,s)-C(\gamma ,t)}{s-t}\right| & \; & \mathrm{for}\;M\mathrm{-a}.e.\;\gamma \in \Gamma . \end{array}$$

Some remarks concerning the uncertain variable k, given by (8) in Definition 8, are in order here. It was proved by Yao, Gao et al. [36] that

(9) $$\begin{array}{cccc} \; & M(\{\gamma \in \Gamma ;\;k(\gamma )⩽x\})⩾2\Phi (x)-1,\; & \; & x\in R_{+}, \end{array}$$

where the function Φ(x), given as in (4), is the uncertainty distribution of a standard normal uncertain variable. By the definition of limit superior of a sequence of sets, we have

$$\begin{array}{cccc} \; & \left\{\gamma \in \Gamma ;\;k\left(\gamma \right)⩽x\right\}\subset \underset{x\to +\infty }{lim sup}\left\{\gamma \in \Gamma ;\;k\left(\gamma \right)⩽x\right\},\; & \; & x\in R_{+}. \end{array}$$

This, together with (9), implies immediately

$$\begin{array}{cc} M(\underset{x\to +\infty }{lim sup}\left\{\gamma \in \Gamma ;\;k\left(\gamma \right)⩽x\right\})⩾ & M(\{\gamma \in \Gamma ;\;k(\gamma )⩽x\})\\ ⩾ & 2\Phi \left(x\right)-1,\;x\in R_{+}, \end{array}$$

which implies further

$$M\left(\underset{x\to +\infty }{lim sup}\left\{\gamma \in \Gamma ;\;k\left(\gamma \right)⩽x\right\}\right)⩾2\lim_{x\to +\infty }\Phi \left(x\right)-1=1.$$

This, alongside with the definition of the uncertainty measure M (see Definition 7), implies

$$M(\underset{x\to +\infty }{lim sup}\left\{\gamma \in \Gamma ;\;k\left(\gamma \right)⩽x\right\})=1.$$

This implies, in particular, that possibly there exists a M-null set in Γ such that, for every sample γ in the sample space Γ, it holds that k(γ)∈[0,+∞) (see (8) for the definition of k).

**Definition 9.**  *Let a, $b\in R_{+}$ with a<b, ${\left\{C\left(t\right)\right\}}_{t\in R_{+}}$ a canonical Liu process, and ${\left\{X\left(t\right)\right\}}_{t\in [a,b]}$ a given L-adapted uncertain process. If there exists an uncertain variable ξ such that*

$$\begin{array}{cccc} \; & \xi =\lim_{∥\Delta ∥\to 0}\sum_{k=0}^{n-1}X\left(t_{k}\right)(C\left(t_{k+1}\right)-C\left(t_{k}\right)),\; & \; & M\mathrm{-a}.s. \end{array}$$

*where Δ: $a=t_{0}<t_{1}<\cdots <t_{n}=b$ is a partition of the compact interval [a,b], and*

$$∥\Delta ∥=\max_{0⩽k⩽n-1}\left(x_{k+1}-x_{k}\right),$$

*then the uncertain process ${\left\{X\left(t\right)\right\}}_{t\in [a,b]}$ is said to be integrable, and the limit uncertain variable ξ is said to be the uncertain integral of ${\left\{X\left(t\right)\right\}}_{t\in [a,b]}$ in the interval [a,b] with respect to the canonical Liu process ${\left\{C\left(t\right)\right\}}_{t\in R_{+}}$. In this situation, we denote*

$$\xi =\int_{a}^{b}X\left(t\right)dC\left(t\right).$$

Suppose that the uncertain process ${\left\{X\left(s\right)\right\}}_{s\in [a,b]}$ is uncertain integrable in [a,b] with respect to the canonical Liu process ${\left\{C\left(s\right)\right\}}_{s\in R_{+}}$. By virtue of Definition 9, we can conclude that for every t∈[a,b], the uncertain process ${\left\{X\left(s\right)\right\}}_{s\in [a,b]}$ is uncertain integrable in the compact subinterval [a,t] with respect to the canonical Liu process ${\left\{C\left(s\right)\right\}}_{s\in R_{+}}$, and that ${\left\{Y\left(t\right)\right\}}_{t\in [a,b]}$ is also an uncertain process with ${\left\{Y\left(t\right)\right\}}_{t\in [a,b]}$ given by

(10) $$\begin{array}{cccc} \; & Y\left(t\right)=\int_{a}^{t}X\left(s\right)dC\left(s\right),\; & \; & t\in [a,b],\;M\mathrm{-a}.s. \end{array}$$

Let *a*, $b\in R_{+}$ with a<b, ${\left\{C\left(t\right)\right\}}_{t\in R_{+}}$ a canonical Liu process, ${\left\{\mu \left(t\right)\right\}}_{t\in [a,b]}$, ${\left\{\sigma \left(t\right)\right\}}_{t\in [a,b]}$ and ${\left\{X\left(t\right)\right\}}_{t\in [a,b]}$ three given L-adapted uncertain processes. If ${\left\{\mu \left(s\right)\right\}}_{s\in [a,b]}$ is M-almost surely Lebesgue integrable in [a,b], ${\left\{\sigma \left(s\right)\right\}}_{s\in [a,b]}$ is uncertain integrable in [a,b] with respect to the canonical Liu process ${\left\{C\left(s\right)\right\}}_{s\in R_{+}}$, and moreover it holds that

$$\begin{array}{cccc} \; & X\left(t\right)=X\left(a\right)+\int_{a}^{t}\mu \left(s\right)ds+\int_{a}^{t}\sigma \left(s\right)dC\left(s\right),\; & \; & t\in [a,b],\;M\mathrm{-a}.s., \end{array}$$

then we call ${\left\{X\left(t\right)\right\}}_{t\in [a,b]}$ a Liu process, and write equivalently

dX(t)=μ(t)dt+σ(t)dC(t),

in which, μ(t)dt and σ(t)dC(t) are called the drift and diffusion terms, respectively.

**Lemma 1**  (see References [16,17])**.** *Let f(x,t) be a $C^{1}$ (by $C^{1}$, we mean the totality of continuous functions whose first order derivative is continuous) function on $R^{2}$, and X(t) a Liu process with μ(t) and σ(t) as its drift and diffusion coefficients, respectively, or equivalently*

(11) $$\begin{array}{cccc} \; & dX(t)=\mu (t)dt+\sigma (t)dC(t),\; & \; & t\in R_{+},\;M\mathrm{-a}.s. \end{array}$$

*Then, f(X(t),t) is a Liu process with $\mu \left(t\right)f_{x}\left(X\left(t\right),t\right)+f_{t}\left(X\left(t\right),t\right)$ and $\sigma \left(t\right)f_{x}\left(X\left(t\right),t\right)$ as its drift term and diffusion term coefficients, respectively, in other words,*

(12) $$\begin{array}{cc} df(X(t),t)= & \left(\mu \left(t\right)f_{x}\left(X\left(t\right),t\right)+f_{t}\left(X\left(t\right),t\right))dt\right)\\ \; & +\sigma \left(t\right)f_{x}\left(X\left(t\right),t\right)dC\left(t\right),\;t\in R_{+},\;M\mathrm{-a}.s. \end{array}$$

**Lemma 2**  (see Reference [18])**.** *Let ${\left\{C\left(t\right)\right\}}_{t\in R_{+}}$ be the aforementioned canonical Liu process, and k the uncertain variable given as in Definition 8 (see *(8)* for the details). For any two constants a and $b\in R_{+}$ with a<b, and any integrable L-adapted uncertain process ${\left\{X\left(t\right)\right\}}_{t\in [a,b]}$, it holds that*

(13) $$\begin{array}{cccc} \; & |\int_{a}^{b}X\left(t\right)dC\left(t\right)|⩽k\int_{a}^{b}\left|X\left(t\right)\right|dt,\; & \; & M\mathrm{-a}.s.\; \end{array}$$

Let *a*, $b\in R_{+}$ with a<b and ${\left\{C\left(t\right)\right\}}_{t\in R_{+}}$ a canonical Liu process. If for every k=1,2,…,N, the uncertain process ${\left\{x_{k}\left(t\right)\right\}}_{t\in [a,b]}$ is uncertain integrable in the interval [a,b] with respect to the canonical Liu process ${\left\{C\left(s\right)\right\}}_{s\in R_{+}}$, then we write

$$\int_{a}^{b}x\left(t\right)dC\left(t\right)={\left(\int_{a}^{b}x_{1}\left(t\right)dC\left(t\right),\int_{a}^{b}x_{2}\left(t\right)dC\left(t\right),\ldots ,\int_{a}^{b}x_{N}\left(t\right)dC\left(t\right)\right)}^{⊤},$$

where $x\left(t\right)={\left(x_{1}\left(t\right),x_{2}\left(t\right),\ldots ,x_{N}\left(t\right)\right)}^{⊤}$. By virtue of this definition, as with (10), we can define the following $R^{N}$-valued uncertain process

$$\begin{array}{cccc} \; & \int_{a}^{t}x\left(s\right)dC\left(s\right),\; & \; & t\in [a,b],\;M-a.s. \end{array}$$

And as with the one-dimensional case, we can define $R^{N}$-valued Liu process. And it is not difficult to imagine that we can establish a counterpart of Lemma 1 for the $R^{N}$-valued Liu process ${\left\{x\left(t\right)\right\}}_{t\in [a,b]}$ given by

dx(t)=μ(t)dt+σ(t)dC(t).

In comparison with this, it seems to be unapparent and therefore much more laborious to establish a counterpart of Lemma 2 for uncertain integrals of $R^{N}$-valued uncertain processes with respect to one dimensional canonical Liu processes.

**Lemma 3.**  *Let ${\left\{C\left(t\right)\right\}}_{t\in R_{+}}$ be the aforementioned canonical Liu process, and k the uncertain variable given as in Definition 8 (see *(8)* for the details). For any two constants a and $b\in R_{+}$ with a<b, and any integrable L-adapted uncertain process ${\left\{x\left(t\right)\right\}}_{t\in [a,b]}$ ($x\left(t\right)={\left(x_{1}\left(t\right),x_{2}\left(t\right),\ldots ,x_{N}\left(t\right)\right)}^{⊤}\in R^{N}$), it holds that*

(14) $$\begin{array}{cccc} \; & \sqrt{(\int_{a}^{b}x\left(t\right)dC\left(t\right){)}^{⊤}\int_{a}^{b}x\left(t\right)dC\left(t\right)}⩽k\int_{a}^{b}\sqrt{x^{⊤}\left(t\right)x\left(t\right)}dt,\; & \; & M\mathrm{-a}.s.\; \end{array}$$

**Proof.**  Thanks to Lemma 2, we have

(15) $$\begin{array}{cccc} (\int_{a}^{b}X\left(t\right)dC\left(t\right){)}^{⊤}\int_{a}^{b}X\left(t\right)dC\left(t\right)= & \sum_{k=1}^{N}(\int_{a}^{b}x_{k}\left(t\right)dC\left(t\right){)}^{2} & \; & \\ ⩽ & k^{2}\sum_{k=1}^{N}(\int_{a}^{b}\left|x_{k}\left(t\right)\right|dt{)}^{2},\; & \; & M\mathrm{-a}.s.\; \end{array}$$

By virtue of some careful calculations, we have further that

(16) $$\begin{array}{cccc} \sum_{k=1}^{N}(\int_{a}^{b}\left|x_{k}\left(t\right)\right|dt{)}^{2}= & \sum_{k=1}^{N}\int_{a}^{b}\left(\int_{a}^{b}|x_{k}\left(t\right)|dt\right)|x_{k}\left(s\right)|ds & \; & \\ = & \int_{a}^{b}\sum_{k=1}^{N}\left(\int_{a}^{b}|x_{k}\left(t\right)|dt\right)|x_{k}\left(s\right)|ds & \; & \\ ⩽ & \sqrt{\sum_{k=1}^{N}(\int_{a}^{b}\left|x_{k}\left(t\right)\right|dt{)}^{2}}\int_{a}^{b}\sqrt{\sum_{k=1}^{N}{\left|x_{k}\left(s\right)\right|}^{2}}ds,\; & \; & M\mathrm{-a}.s., \end{array}$$

in which, the ‘⩽’ follows from the well-known Cauchy–Schwarz inequality. On the other hand, we can deduce from (16) immediately that

(17) $$\begin{array}{cc} \sqrt{\sum_{k=1}^{N}(\int_{a}^{b}\left|x_{k}\left(t\right)\right|dt{)}^{2}}⩽ & \int_{a}^{b}\sqrt{\sum_{k=1}^{N}{\left|x_{k}\left(s\right)\right|}^{2}}ds\\ = & \int_{a}^{b}\sqrt{x^{⊤}\left(t\right)x\left(t\right)}dt,\;M\mathrm{-a}.s.\; \end{array}$$

Plug (17) into (15), and conduct some easy calculations, to end the proof of Lemma 3. □

It is worth pointing that, as can be seen from (8) in Definition 8, the uncertain variable k depends merely on the aforementioned canonical Liu process ${\left\{C\left(t\right)\right\}}_{t\in R_{+}}$, in particular, k is independent of *a*, *b* and the uncertain process ${\left\{X\left(t\right)\right\}}_{t\in [a,b]}$.

Let $A\in R^{N\times N}$ be a positive definite matrix. Then, by the well-known theorem of Linear Algebra, *A* admits a unique Cholesky decomposition, that is, there exists a unique a upper triangular matrix ⨿ with positive diagonal entries such that $A={⨿}^{⊤}⨿$. Hereafter, we shall write ${⨿}_{A}$ for the unique aforementioned upper triangular matrix in the Cholesky decomposition of any positive definite matrix *A*; It is straightforward to see that the matrix ${⨿}_{A}$ is nonsingular. With the help of the notion of Cholesky decomposition, we can prepare the following lemma which is extremely useful in our later presentation.

**Lemma 4.**  *Let $A\in R^{N\times N}$ be a positive definite matrix, and $B\in R^{N\times N}$ a positive semi-definite matrix. Then, the following two identities hold true:*

(18) $$\max_{x\in R^{N}\backslash \left\{0\right\}}\frac{x^{⊤}Bx}{x^{⊤}Ax}=\lambda_{\max}\left({\left({⨿}_{A}^{-1}\right)}^{⊤}B{⨿}_{A}^{-1}\right),$$

*and*

$$\min_{x\in R^{N}\backslash \left\{0\right\}}\frac{x^{⊤}Bx}{x^{⊤}Ax}=\lambda_{\min}\left({\left({⨿}_{A}^{-1}\right)}^{⊤}B{⨿}_{A}^{-1}\right).$$

**Proof.**  It is obvious that the matrix ${\left({⨿}_{A}^{-1}\right)}^{⊤}B{⨿}_{A}^{-1}$ is positive definite (a fortiori, symmetric). By Jordan’s decomposition theorem, there exists an orthogonal matrix *Q* such that

$${\left({⨿}_{A}^{-1}\right)}^{⊤}B{⨿}_{A}^{-1}=Q^{⊤}\mathrm{diag}\left(\lambda_{1},\lambda_{2},\ldots ,\lambda_{N}\right)Q.$$

Pre- and post-multiply both sides of this equation by ${\left({⨿}_{A}\right)}^{⊤}$ and ${⨿}_{A}$, respectively, to obtain

$$B={\left({⨿}_{A}\right)}^{⊤}Q^{⊤}\mathrm{diag}\left(\lambda_{1},\lambda_{2},\ldots ,\lambda_{N}\right)Q{⨿}_{A},$$

where $\lambda_{k}⩾0$ (k=1,2,…,N). This, together with the definition of ${⨿}_{A}$, implies

(19) $$\frac{x^{⊤}Bx}{x^{⊤}Ax}=\frac{x^{⊤}{\left({⨿}_{A}\right)}^{⊤}Q^{⊤}\mathrm{diag}\left(\lambda_{1},\lambda_{2},\ldots ,\lambda_{N}\right)Q{⨿}_{A}x}{x^{⊤}{\left({⨿}_{A}\right)}^{⊤}Q^{⊤}Q{⨿}_{A}x}.$$

Aided by (19), we can complete the proof of Lemma 4, via some routine calculations. □

**Lemma 5.**  *Let $P\in R^{N\times N}$ be a positive definite matrix.*

*(Jensen’s inequality). Let a, b∈R be any two constants with a<b. For any square integrable vector-valued function $\left[a,b\right]∋t\mapsto y\left(t\right)\in R^{N}$ in Lebesgue’s sense, it holds that*

(20) $$(\int_{a}^{b}y\left(t\right)dt{)}^{⊤}P\int_{a}^{b}y\left(t\right)dt⩽\left(b-a\right)\int_{a}^{b}y^{⊤}\left(t\right)Py\left(t\right)dt,$$

*(Extrema of Rayleigh’s quotient). It holds always that*

(21) $$\begin{array}{cccc} \; & x^{⊤}Px⩾\lambda_{\min}\left(P\right)x^{⊤}x,\; & \; & x\in R^{N}, \end{array}$$

*and that*

(22) $$\begin{array}{cccc} \; & x^{⊤}Py⩽\lambda_{\max}\left(P\right)\sqrt{\left(x^{⊤}x\right)\left(y^{⊤}y\right)},\; & \; & x,y\in R^{N}. \end{array}$$

### 2.2. Formulation of the Problems and Main Assumptions

In this paper, we consider the model DUCNNs

(23) $$\left\{\begin{array}{c} \begin{array}{cc} dx(t)= & (-Dx\left(t\right)+A_{0}F_{0}\circ \left(x\left(t\right)\right)+A_{1}F_{1}\circ \left(x\left(t-\tau_{1}\right)\right)\\ \; & +A_{2}\int_{t-\tau_{2}}^{t}F_{2}\circ \left(x\left(s\right)\right)ds+U\left(t\right))dt\\ \; & +(B_{0}G_{0}\circ \left(x\left(t\right),t\right)+B_{1}G_{1}\circ \left(x\left(t-\eta_{1}\right),t\right)\\ \; & +B_{2}\int_{t-\eta_{2}}^{t}G_{2}\circ \left(x\left(s\right),t\right)ds+V\left(t\right))dC\left(t\right),\;t\in R_{+},\;M\mathrm{-a}.s., \end{array}\\ x\left(t\right)=x_{0}\left(t\right),\;t\in \left[-\max\left(\tau_{1},\tau_{2},\eta_{1},\eta_{2}\right),0\right],\;M\mathrm{-a}.s., \end{array}\right.$$

in which: x(t) is a state trajectory and can be re-written in component form as

$$\begin{array}{cccc} \; & x\left(t\right)={\left(x_{1}\left(t\right),x_{2}\left(t\right),\ldots ,x_{N}\left(t\right)\right)}^{⊤},\; & \; & t\in R_{+},\;M\mathrm{-a}.s.; \end{array}$$

the matrix $D=\mathrm{diag}(d_{1},d_{2},\ldots ,d_{N})$ in the leakage term −Dx(t) is positive definite; $A_{k}$ and $B_{k}$ are the connection weight coefficient matrices (real square matrices) in the transmission terms, k=0,1,2; the activation functions $F_{k}$ and $G_{k}$ can be written in component form as

(24) $$\begin{array}{cccc} \; & F_{k}\circ \left(x\right)={\left(f_{k1}\left(x_{1}\right),f_{k2}\left(x_{2}\right),\ldots ,f_{kN}\left(x_{N}\right)\right)}^{⊤},\; & \; & x={\left(x_{1},x_{2},\ldots ,x_{N}\right)}^{⊤}\in R^{N} \end{array}$$

and

(25) $$\begin{array}{cccc} \; & G_{k}\circ \left(x\right)={\left(g_{k1}\left(x_{1}\right),g_{k2}\left(x_{2}\right),\ldots ,g_{kN}\left(x_{N}\right)\right)}^{⊤},\; & \; & x={\left(x_{1},x_{2},\ldots ,x_{N}\right)}^{⊤}\in R^{N}, \end{array}$$

respectively, k=0,1,2; the positive constants $\tau_{1}$, $\tau_{2}$, $\eta_{1}$ and $\eta_{2}$ are the time delay; as stated previously, ${\left\{C\left(t\right)\right\}}_{t\in R_{+}}$ is a canonical Liu process on the uncertainty space (Γ,L,L,M); the initial state $x_{0}:\Gamma \times \left[-\max\left(\tau_{1},\tau_{2},\eta_{1},\eta_{2}\right),0\right]$ is $L_{0}⊗L$ measurable.

**Definition 10.**  A $L_{0}$-measurable N dimensional uncertain variable $x^{*}$ is said to be a equilibrium state (or fixed point) of the model DUCNNs (23)*, provided that*

(26) $$\begin{array}{cccc} \; & -Dx^{*}+A_{0}F_{0}\circ \left(x^{*}\right)+A_{1}F_{1}\circ \left(x^{*}\right)+\tau_{2}A_{2}F_{2}\circ \left(x^{*}\right)+U\left(t\right)=0, & \; & t\in R_{+},\;M\mathrm{-a}.s., \end{array}$$

*and that*

(27) $$\begin{array}{cccc} \; & B_{0}G_{0}\circ \left(x^{*},t\right)+B_{1}G_{1}\circ \left(x^{*},t\right)+\eta_{2}B_{2}G_{2}\circ \left(x^{*},t\right)+V\left(t\right)=0, & \; & t\in R_{+},\;M\mathrm{-a}.s. \end{array}$$

**Definition 11.**  Suppose that the uncertain variable $x^{*}:\Gamma \to R^{N}$, required to be $L_{0}$-measurable, is a equilibrium state (or fixed point) of the model DUCNNs (23). $x^{*}$ is said to be M-a.s. exponentially stable provided that there exists a positive definite matrix $P\in R^{N\times N}$, as well as two uncertain variables ι:Γ→[0,+∞) and 𝚥:Γ→(0,+∞) such that for any state trajectory x(t) of DUCNNs (23)*, it holds that*

(28) $$\begin{array}{cccc} \; & {\left(x\left(t\right)-x^{*}\right)}^{⊤}P\left(x\left(t\right)-x^{*}\right)⩽\iota e^{-𝚥t},\; & \; & t\in R_{+},\;M\mathrm{-a}.s.\; \end{array}$$

From the perspectives of the mathematical complexity and application, it seems to be more interesting to require the decaying exponent 𝚥 in (28) (see Definition 11) be essentially bounded, or equivalently, to require 𝚥 be an absolute positive constant.

By some routine but seemingly tedious calculations, we can conclude that the decay estimate (28) in Definition 11 holds true if and only if the assertion holds true: either (i) there exists a positive time instant $T^{*}$ such that

(29) $$\begin{array}{cccc} \; & {\left(x\left(t\right)-x^{*}\right)}^{⊤}P\left(x\left(t\right)-x^{*}\right)=0,\; & \; & t\in [T^{*},+\infty ),\;M\mathrm{-a}.s.\; \end{array}$$

which is equivalent to

(30) $$\begin{array}{cccc} \; & x\left(t\right)=x^{*},\; & \; & t\in [T^{*},+\infty ),\;M\mathrm{-a}.s.\;, \end{array}$$

or (ii) $x\left(t\right)\neq x^{*}$ for every $t\in R_{+}$ and

(31) $$\begin{array}{cccc} \; & \underset{t\to +\infty }{lim sup}\frac{\ln\left({\left(x\left(t\right)-x^{*}\right)}^{⊤}P\left(x\left(t\right)-x^{*}\right)\right)}{t}⩽-𝚥, & \; & M\mathrm{-a}.s. \end{array}$$

Based on the analysis conducted in this paragraph, we conclude that proving that the equilibrium state (or fixed point) of DUCNNs (23) boils down to proving the inequality (31) holds true under the assumption that $x\left(t\right)\neq x^{*}$ for any $t\in R_{+}$. The positive valued uncertain variable 𝚥 in (28) and (31) is called a (exponential) decay rate.

**Assumption 1.**  *The L-adapted uncertain processes ${\left\{U\left(t\right)\right\}}_{t\in R_{+}}$ and ${\left\{V\left(t\right)\right\}}_{t\in R_{+}}$ satisfy*

$$\begin{array}{cccc} \; & U(t)\equiv U(0)\;\mathrm{and}\;V(t)\equiv V(0),\; & \; & t\in R_{+},\;M\mathrm{-a}.s.\; \end{array}$$

The ($L_{0}$-measurable) uncertain variable $x^{*}:\Gamma \to R^{N}$ satisfies automatically (27), M almost surely, whenever $x^{*}$ is a solution to (26)*.*

**Assumption 2.**  *The constants $\tau_{k}$ and $\eta_{k}$, independent of sample and time, (occurred in the model DUCNNs (23)) are all non-negative, k=1,2. Moreover, it holds always that*

$$\tau_{2}=\max\left(\tau_{1},\;\tau_{2},\;\eta_{1},\;\eta_{2}\right).$$

**Assumption 3.**  *The activation function $F_{k}$ is Lipschitz continuous and satisfies the linear growth condition at infinity, k=0, 1, 2. More precisely, it holds that*

(32) $$\begin{array}{cccc} \; & \mathrm{tr}\left({\left(L_{F_{k}}\right)}^{2}\right)<+\infty , & \; & k=0,1,2, \end{array}$$

*where $L_{F_{k}}$ is a diagonal matrix defined by*

(33) $$\begin{array}{cccc} \; & L_{F_{k}}=\mathrm{diag}\left(l_{F_{k}}^{1},l_{F_{k}}^{2},\ldots ,l_{F_{k}}^{N}\right), & \; & k=0,1,2, \end{array}$$

*with the diagonal entry $l_{F_{k}}^{j}$, a non-negative constant, given by*

(34) $$\begin{array}{cccc} \; & l_{F_{k}}^{j}=\underset{x,y\in R,\;x\neq y}{\sup}\frac{|f_{kj}\left(x\right)-f_{kj}\left(y\right)|}{\left|x-y\right|}, & \; & j=1,2,\ldots ,N,\;k=0,1,2. \end{array}$$

*The activation function $G_{k}$ satisfies Carathéodory’s condition, is Lipschitz continuous and satisfies linear growth condition at infinity, k=0, 1, 2. In addition, it holds that*

(35) $$\begin{array}{cccc} \; & \int_{0}^{+\infty }\mathrm{tr}\left({\left(L_{G_{k}}\left(s\right)\right)}^{2}\right)ds<+\infty , & \; & k=0,1,2, \end{array}$$

*where the diagonal matrix $L_{G_{k}}\left(s\right)$, as with the matrix $L_{F_{k}}$, assumes the form*

(36) $$\begin{array}{cccc} \; & L_{G_{k}}\left(s\right)=\mathrm{diag}\left(l_{G_{k}}^{1}\left(s\right),l_{G_{k}}^{2}\left(s\right),\ldots ,l_{G_{k}}^{N}\left(s\right)\right), & \; & k=0,1,2, \end{array}$$

*with the function $l_{G_{k}}^{j}\left(s\right)$, defined in the interval $R_{+}$, being Lebesgue integrable in $R_{+}$, being essentially bounded in $R_{+}$ and defined explicitly by*

(37) $$\begin{array}{cccc} \; & l_{G_{k}}^{j}\left(s\right)=\underset{x,y\in R,\;x\neq y}{\sup}\frac{|g_{kj}\left(x,s\right)-G_{kj}\left(y,s\right)|}{\left|x-y\right|}, & \; & j=1,2,\ldots ,N,\;k=0,1,2. \end{array}$$

## 3. Main Results and the Proofs

**Theorem 1.**  *Suppose that Assumptions 1 and 3 hold true. DUCNNs *(23)* admit unique equilibrium states (or fixed points), provided ς<1 with the non-negative constant ς defined by*

(38) $$\begin{array}{cc} \varsigma = & \frac{1}{\lambda_{\min}\left(D\right)}(\sqrt{\lambda_{\max}\left(A_{0}^{⊤}A_{0}\right)}\lambda_{\max}\left(L_{F_{0}}\right)+\sqrt{\lambda_{\max}\left(A_{1}^{⊤}A_{1}\right)}\lambda_{\max}\left(L_{F_{1}}\right)\\ \; & +\tau_{2}\sqrt{\lambda_{\max}\left(A_{2}^{⊤}A_{2}\right)}\lambda_{\max}\left(L_{F_{2}}\right)). \end{array}$$

**Proof.**  Let us recall that $R^{N}$, equipped with the mapping

$$R^{N}∋x\mapsto \sqrt{x^{⊤}x}\in R_{+},$$

is indeed a Banach space, and the natural induced metric space is complete.

Let us write, in this proof, U:−U(0) (it is worth reminding that U(0)≡U(t) for every $t\in R_{+}$, M-a.s.). Since *D* is positive definite, it is non-singular. This implies, in particular, that for any ***x***, there exists a unique Λ(x) such that

(39) $$D\Lambda \left(x\right)=A_{0}F_{0}\circ \left(x\right)+A_{1}F_{1}\circ \left(x\right)+\tau_{2}A_{2}F_{2}\circ \left(x\right)+U.$$

Thus, we obtain a mapping Λ of $R^{N}$ into itself. For any $x_{1}$ and $x_{2}$, we have

$$D\Lambda \left(x_{1}\right)=A_{0}F_{0}\circ \left(x_{1}\right)+A_{1}F_{1}\circ \left(x_{1}\right)+\tau_{2}A_{2}F_{2}\circ \left(x_{1}\right)+U,$$

and

$$D\Lambda \left(x_{2}\right)=A_{0}F_{0}\circ \left(x_{2}\right)+A_{1}F_{1}\circ \left(x_{2}\right)+\tau_{2}A_{2}F_{2}\circ \left(x_{2}\right)+U.$$

We have therefore

$$\begin{array}{cc} D(\Lambda \left(x_{2}\right)-\Lambda \left(x_{1}\right))= & A_{0}\left(F_{0}\circ \left(x_{2}\right)-F_{0}\circ \left(x_{1}\right)\right)+A_{1}\left(F_{1}\circ \left(x_{2}\right)-F_{1}\circ \left(x_{1}\right)\right)\\ \; & +\tau_{2}A_{2}\left(F_{2}\circ \left(x_{2}\right)-F_{2}\circ \left(x_{1}\right)\right), \end{array}$$

which implies further that

$$\begin{array}{cc} \lambda_{\min}\left(D\right)\sqrt{{\left(\Lambda \left(x_{2}\right)-\Lambda \left(x_{1}\right)\right)}^{⊤}\left(\Lambda \left(x_{2}\right)-\Lambda \left(x_{1}\right)\right)}⩽ & \sqrt{{\left(\Lambda \left(x_{2}\right)-\Lambda \left(x_{1}\right)\right)}^{⊤}D^{2}\left(\Lambda \left(x_{2}\right)-\Lambda \left(x_{1}\right)\right)}\\ ⩽ & \sqrt{\left(F_{0}\circ \left(x_{2}\right)-F_{0}\circ \left(x_{1}\right)\right)A_{0}^{⊤}A_{0}\left(F_{0}\circ \left(x_{2}\right)-F_{0}\circ \left(x_{1}\right)\right)}\\ \; & +\sqrt{\left(F_{1}\circ \left(x_{2}\right)-F_{1}\circ \left(x_{1}\right)\right)A_{1}^{⊤}A_{1}\left(F_{1}\circ \left(x_{2}\right)-F_{1}\circ \left(x_{1}\right)\right)}\\ \; & +\tau_{2}\sqrt{\left(F_{2}\circ \left(x_{2}\right)-F_{2}\circ \left(x_{1}\right)\right)A_{2}^{⊤}A_{2}\left(F_{2}\circ \left(x_{2}\right)-F_{2}\circ \left(x_{1}\right)\right)}\\ ⩽ & (\sqrt{\lambda_{\max}\left(A_{0}^{⊤}A_{0}\right)}\lambda_{\max}\left(L_{F_{0}}\right)+\sqrt{\lambda_{\max}\left(A_{1}^{⊤}A_{1}\right)}\lambda_{\max}\left(L_{F_{1}}\right)\\ \; & +\tau_{2}\sqrt{\lambda_{\max}\left(A_{2}^{⊤}A_{2}\right)}\lambda_{\max}\left(L_{F_{2}}\right))\sqrt{{\left(x_{2}-x_{1}\right)}^{⊤}\left(x_{2}-x_{1}\right)}. \end{array}$$

Recalling the notation ς defined by (38), we conclude immediately

(40) $$\sqrt{{\left(\Lambda \left(x_{2}\right)-\Lambda \left(x_{1}\right)\right)}^{⊤}\left(\Lambda \left(x_{2}\right)-\Lambda \left(x_{1}\right)\right)}⩽\varsigma \sqrt{{\left(x_{2}-x_{1}\right)}^{⊤}\left(x_{2}-x_{1}\right)}.$$

By Banach’s fixed-point theorem, this, together with the assumption that ς<1, implies that Λ admits a unique fixed point $x^{*}$. Recalling (39), we conclude that $\Lambda \left(x^{*}\right)=x^{*}$ implies that $x^{*}$ satisfies (26), and furthermore satisfies automatically (27). Since *D* is non-singular (positive definite, actually), the activation function $F_{k}$ is globally Lipschitz continuous (k=0,1,2), and *U* is $L_{0}$-measurable, $x^{*}$ is $L_{0}$-measurable. By Definition 10, $x^{*}$ is indeed a a equilibrium state (or fixed point) of the model DUCNNs (23).

Assume that $x_{1}^{*}$ and $x_{2}^{*}$ are equilibrium states (or fixed points) of DUCNNs (23). By the above analysis, $x_{1}^{*}$ and $x_{2}^{*}$ are fixed points of Λ. In view of (40), we have

$$\begin{array}{cc} \sqrt{{\left(x_{2}-x_{1}\right)}^{⊤}\left(x_{2}-x_{1}\right)}= & \sqrt{{\left(\Lambda \left(x_{2}\right)-\Lambda \left(x_{1}\right)\right)}^{⊤}\left(\Lambda \left(x_{2}\right)-\Lambda \left(x_{1}\right)\right)}\\ ⩽ & \varsigma \sqrt{{\left(x_{2}-x_{1}\right)}^{⊤}\left(x_{2}-x_{1}\right)}. \end{array}$$

Noting that ς<1, we conclude that $x_{2}-x_{1}=0$, or equivalently, $x_{1}=x_{2}$.

In conclusion, the proof of Theorem 1 is complete. □

**Theorem 2.**  *Suppose that Assumptions 1, 2 and 3 hold true. If the non-negative constant ς given by *(38)* is strictly less than 1, there exists a positive definite matrix $\Phi \in R^{N\times N}$, four positive definite matrices $\Psi_{1}\in R^{N\times N}$, $\Psi_{2}\in R^{N\times N}$, $\Psi_{3}\in R^{N\times N}$ as well as $\Omega \in R^{N\times N}$, and three positive constants $\delta_{1}$, $\delta_{2}$ alongside with $\delta_{3}$ such that*

(41) $$\Psi_{2}-\delta_{2}\Phi B_{1}B_{1}^{⊤}\Phi ≻0\left(\in R^{N\times N}\right),$$

*and*

(42) $$\Theta =\left(\begin{array}{ccc} ℧ & -\Phi A_{1} & -\Phi A_{2}\\ {}* & \Psi_{1} & 0\\ {}* & * & \Omega \end{array}\right)≻0\left(\in R^{\left(3N)\times (3N\right)}\right),$$

*where the symmetric matrix (can be proved to be positive definite) ℧ is given by*

$$\begin{array}{cc} ℧= & \mathrm{sym}\left(\Phi D\right)-\Phi A_{0}A_{0}^{⊤}\Phi^{⊤}-{\left(L_{F_{0}}\right)}^{2}-\lambda_{\max}\left(\Psi_{1}\right)L_{F_{1}}\\ \; & -\Psi_{2}-\Psi_{3}-\tau_{2}\Omega -\delta_{1}\Phi B_{0}B_{0}^{⊤}\Phi -\delta_{3}\eta_{2}\Phi B_{2}B_{2}^{⊤}\Phi , \end{array}$$

*then DUCNNs *(23)* have unique equilibrium states (or fixed points), and the equilibrium states (or fixed points) are almost surely exponentially stable at a decay rate κ given by*

(43) $$\begin{array}{cc} \frac{1}{\kappa }=\max( & \lambda_{\max}\left({\left({⨿}_{\Theta }^{-1}\right)}^{⊤}\tilde{\Phi }{⨿}_{\Theta }^{-1}\right),\;\lambda_{\max}\left(\Psi_{1}\right)\lambda_{\max}\left({\left({⨿}_{\Omega }^{-1}\right)}^{⊤}{\left(L_{F_{1}}\right)}^{2}{⨿}_{\Omega }^{-1}\right)\\ \; & +\lambda_{\max}\left({\left({⨿}_{\Omega }^{-1}\right)}^{⊤}\Psi_{2}{⨿}_{\Omega }^{-1}\right)+\lambda_{\max}\left({\left({⨿}_{\Omega }^{-1}\right)}^{⊤}\Psi_{3}{⨿}_{\Omega }^{-1}\right)+\tau_{2}), \end{array}$$

*where the matrix $\tilde{\Phi }\in R^{\left(3N)\times (3N\right)}$ is given by*

(44) $$\tilde{\Phi }=\left(\begin{array}{ccc} \Phi & 0 & 0\\ {}* & 0 & 0\\ {}* & * & 0 \end{array}\right).$$

**Proof.**  In view of the assumption that ς<1 (see (38)), by Theorem 1, we conclude that DUCNNs (23) have unique equilibrium states (or fixed points). It remains to prove the almost surely exponential stability part of Theorem 2. In the rest of this proof, we write $x^{*}$ for an equilibrium state (or fixed point) of DUCNNs (23). As remarked previously, to prove Theorem 2, it suffices to establish the inequality (31) for every state trajectory x(t) of DUCNNs (31) fulfilling $x\left(t\right)\neq x^{*}$ ($t\in R_{+}$).

For the sake of convenience of our later presentation, we introduce

(45) $$\begin{array}{cccc} \; & w\left(t\right)=x\left(t\right)-x^{*},\; & \; & t\in [-\max\left(\tau_{1},\tau_{2},\eta_{1},\eta_{2}\right),+\infty ),\;M\mathrm{-a}.s., \end{array}$$

and consider the new DUCNNs

(46) $$\left\{\begin{array}{c} \begin{array}{cc} dw(t)= & \left(-Dw\left(t\right)+A_{0}{\overset{ˇ}{F}}_{0}\circ \left(w\left(t\right)\right)+A_{1}{\overset{ˇ}{F}}_{1}\circ \left(w\left(t-\tau_{1}\right)\right)\right.\\ \; & \left.+A_{2}\int_{t-\tau_{2}}^{t}{\overset{ˇ}{F}}_{2}\circ \left(w\left(s\right)\right)ds\right)dt\\ \; & +(B_{0}{\overset{ˇ}{G}}_{0}\circ \left(w\left(t\right),t\right)+B_{1}{\overset{ˇ}{G}}_{1}\circ \left(w\left(t-\eta_{1}\right),t\right)\\ \; & +B_{2}\int_{t-\eta_{2}}^{t}{\overset{ˇ}{G}}_{2}\circ \left(w\left(s\right),t\right)ds)dC\left(t\right),\;t\in R_{+},\;M\mathrm{-a}.s., \end{array}\\ w\left(t\right)=w_{0}\left(t\right),\;t\in \left[-\max\left(\tau_{1},\tau_{2},\eta_{1},\eta_{2}\right),0\right],\;M\mathrm{-a}.s. \end{array}\right.$$

where the initial datum $w_{0}\left(t\right)$ is given by

$$\begin{array}{cccc} \; & w_{0}\left(t\right)=x_{0}\left(t\right)-x^{*},\; & \; & t\in [-\max\left(\tau_{1},\tau_{2},\eta_{1},\eta_{2}\right),0],\;M\mathrm{-a}.s., \end{array}$$

${\overset{ˇ}{F}}_{k}\circ \left(w\left(t\right)\right)$ is given by

(47) $$\begin{array}{cc} {\overset{ˇ}{F}}_{k}\circ \left(w\left(t\right)\right)= & F_{k}\circ \left(x\left(t\right)\right)-F_{k}\circ \left(x^{*}\right)\\ = & F_{k}\circ \left(x^{*}+w\left(t\right)\right)-F_{k}\circ \left(x^{*}\right)\\ = & ({\overset{ˇ}{f}}_{k1}\left(w_{1}\left(t\right)\right),{\overset{ˇ}{f}}_{k2}\left(w_{2}\left(t\right)\right),\ldots ,{\overset{ˇ}{f}}_{kN}\left(w_{N}\left(t\right)\right){)}^{⊤}\\ = & (f_{k1}\left(x_{1}\left(t\right)\right)-f_{k1}\left(x_{1}^{*}\right),f_{k2}\left(x_{2}\left(t\right)\right)-f_{k2}\left(x_{2}^{*}\right),\ldots ,f_{kN}\left(x_{N}\left(t\right)\right)-f_{kN}\left(x_{N}^{*}\right){)}^{⊤}\\ = & (f_{k1}\left(x_{1}^{*}+w_{1}\left(t\right)\right)-f_{k1}\left(x_{1}^{*}\right),f_{k2}\left(x_{2}^{*}+w_{2}\left(t\right)\right)-f_{k2}\left(x_{2}^{*}\right),\ldots ,\\ \; & \;f_{kN}\left(x_{N}^{*}+w_{N}\left(t\right)\right)-f_{kN}\left(x_{N}^{*}\right){)}^{⊤},\;t\in \left[-\max\left(\tau_{1},\tau_{2},\eta_{1},\eta_{2}\right),+\infty \right),\;M\mathrm{-a}.s.,\;k=0,1,2, \end{array}$$

${\overset{ˇ}{G}}_{0}\circ \left(w\left(t\right),t\right)$ is given by

$$\begin{array}{cc} {\overset{ˇ}{G}}_{0}\circ \left(w\left(t\right),t\right)= & G_{k}\circ \left(x\left(t\right),t\right)-G_{k}\circ \left(x^{*},t\right)\\ = & G_{k}\circ \left(x^{*}+w\left(t\right),t\right)-G_{k}\circ \left(x^{*},t\right)\\ = & ({\overset{ˇ}{g}}_{k1}\left(w_{1}\left(t\right),t\right),{\overset{ˇ}{g}}_{k2}\left(w_{2}\left(t\right),t\right),\ldots ,{\overset{ˇ}{g}}_{kN}\left(w_{N}\left(t\right),t\right){)}^{⊤}\\ = & (g_{k1}\left(x_{1}\left(t\right),t\right)-g_{k1}\left(x_{1}^{*},t\right),g_{k2}\left(x_{2}\left(t\right),t\right)-g_{k2}\left(x_{2}^{*},t\right),\ldots ,g_{kN}\left(x_{N}\left(t\right),t\right)-g_{kN}\left(x_{N}^{*},t\right){)}^{⊤}\\ = & (g_{k1}\left(x_{1}^{*}+w_{1}\left(t\right),t\right)-g_{k1}\left(x_{1}^{*},t\right),g_{k2}\left(x_{2}^{*}+w_{2}\left(t\right),t\right)-g_{k2}\left(x_{2}^{*},t\right),\ldots ,\\ \; & \;g_{kN}\left(x_{N}^{*}+w_{N}\left(t\right),t\right)-g_{kN}\left(x_{N}^{*},t\right){)}^{⊤},\;t\in \left[-\max\left(\tau_{1},\tau_{2},\eta_{1},\eta_{2}\right),+\infty \right),\;M\mathrm{-a}.s., \end{array}$$

and ${\overset{ˇ}{G}}_{k}\circ \left(w\left(s\right),t\right)$ is given by

$$\begin{array}{cc} {\overset{ˇ}{G}}_{k}\circ \left(w\left(s\right),t\right)= & G_{k}\circ \left(x\left(s\right),t\right)-G_{k}\circ \left(x^{*},t\right)\\ = & G_{k}\circ \left(x^{*}+w\left(s\right),t\right)-G_{k}\circ \left(x^{*},t\right)\\ = & ({\overset{ˇ}{g}}_{k1}\left(w_{1}\left(s\right),t\right),{\overset{ˇ}{g}}_{k2}\left(w_{2}\left(s\right),t\right),\ldots ,{\overset{ˇ}{g}}_{kN}\left(w_{N}\left(s\right),t\right){)}^{⊤}\\ = & (g_{k1}\left(x_{1}\left(s\right),t\right)-g_{k1}\left(x_{1}^{*},t\right),g_{k2}\left(x_{2}\left(s\right),t\right)-g_{k2}\left(x_{2}^{*},t\right),\ldots ,g_{kN}\left(x_{N}\left(s\right),t\right)-g_{kN}\left(x_{N}^{*},t\right){)}^{⊤}\\ = & (g_{k1}\left(x_{1}^{*}+w_{1}\left(s\right),t\right)-g_{k1}\left(x_{1}^{*},t\right),g_{k2}\left(x_{2}^{*}+w_{2}\left(s\right),t\right)-g_{k2}\left(x_{2}^{*},t\right),\ldots ,\\ \; & \;g_{kN}\left(x_{N}^{*}+w_{N}\left(s\right),t\right)-g_{kN}\left(x_{N}^{*},t\right){)}^{⊤},\;s,t\in \left[-\max\left(\tau_{1},\tau_{2},\eta_{1},\eta_{2}\right),+\infty \right)\;\mathrm{with}\;s⩽t,\;M\mathrm{-a}.s.,\;k=1,2. \end{array}$$

The stability of the equilibrium state (or fixed point) $x^{*}$ of DUCNNs (31) is equivalent to that of the equilibrium state (or fixed point) 0 of DUCNNs (46). The time delay in DUCNNs (31) (or equivalently, in DUCNNs (46)) brings about extreme difficulty in the stability analysis procedure. To overcome the aforementioned difficulty, our basic idea is to make full use of a certain Lyapunov–Krasovskii functional, associated to DUCNNs (46), to take in the after-effect in DUCNNs (31) (or equivalently, in DUCNNs (46)). Let us introduce the positive definite functional for DUCNNs (46)

(48) $$\begin{array}{cccc} \; & V^{\varepsilon }\left(w,t\right)=e^{-\varepsilon t}+V\left(w,t\right), & \; & t\in R_{+},\;M\mathrm{-a}.s. \end{array}$$

in which the positive parameter ε will be chosen appropriately (actually, the parameter ε will be specified deliberately to be equal to κ with the constant κ given ‘implicitly’ by (43)), and V(w,t) is a Lyapunov–Krasovskii functional candidate and can be expressed as

(49) $$\begin{array}{cccc} \; & V\left(w,t\right)=\sum_{k=1}^{3}V_{k}\left(t\right), & \; & t\in R_{+},\;M\mathrm{-a}.s. \end{array}$$

where the functionals $V_{1}\left(t\right)$, $V_{2}\left(t\right)$ and $V_{3}\left(t\right)$ are given, respectively, by

(50) $$\begin{array}{cc} V_{1}\left(t\right)= & {\left(x\left(t\right)-x^{*}\right)}^{⊤}\Phi \left(x\left(t\right)-x^{*}\right)\\ = & w^{⊤}\left(t\right)\Phi w\left(t\right),\;t\in R_{+},\;M\mathrm{-a}.s., \end{array}$$

(51) $$\begin{array}{cc} V_{2}\left(t\right)= & \int_{t-\tau_{1}}^{t}{\left(F_{1}\left(x\left(s\right)\right)-F_{1}\left(x^{*}\right)\right)}^{⊤}\Psi_{1}\left(F_{1}\left(x\left(s\right)\right)-F_{1}\left(x^{*}\right)\right)ds\\ \; & +\int_{t-\eta_{1}}^{t}{\left(x\left(s\right)-x^{*}\right)}^{⊤}\Psi_{2}\left(x\left(s\right)-x^{*}\right)ds\\ \; & +\int_{t-\eta_{2}}^{t}{\left(x\left(s\right)-x^{*}\right)}^{⊤}\Psi_{3}\left(x\left(s\right)-x^{*}\right)ds\\ = & \int_{t-\tau_{1}}^{t}{\left({\overset{ˇ}{F}}_{1}\circ \left(w\left(s\right)\right)\right)}^{⊤}\Psi_{1}{\overset{ˇ}{F}}_{1}\circ \left(w\left(s\right)\right)ds+\int_{t-\eta_{1}}^{t}w^{⊤}\left(s\right)\Psi_{2}w\left(s\right)ds\\ \; & +\int_{t-\eta_{2}}^{t}w^{⊤}\left(s\right)\Psi_{3}w\left(s\right)ds,\;t\in R_{+},\;M\mathrm{-a}.s. \end{array}$$

and

(52) $$\begin{array}{cc} V_{3}\left(t\right)= & \int_{t-\tau_{2}}^{t}\int_{s}^{t}{\left(x\left(\zeta \right)-x^{*}\right)}^{⊤}\Omega \left(x\left(\zeta \right)-x^{*}\right)d\zeta ds\\ = & \int_{t-\tau_{2}}^{t}\int_{s}^{t}w^{⊤}\left(\zeta \right)\Omega w\left(\zeta \right)d\zeta ds,\;t\in R_{+},\;M\mathrm{-a}.s. \end{array}$$

By the chain rule of differentiation for Liu processes (see Lemma 1), we have

(53) $$\begin{array}{cccc} \; & d\ln V^{\varepsilon }\left(w,t\right)=\frac{dV^{\varepsilon }\left(w,t\right)}{V^{\varepsilon }\left(w,t\right)}=\frac{dV\left(w,t\right)-\varepsilon e^{-\varepsilon t}dt}{V^{\varepsilon }\left(w,t\right)}, & \; & t\in R_{+},\;M\mathrm{-a}.s. \end{array}$$

Taking into account of (49), we have immediately

(54) $$\begin{array}{cccc} \; & dV\left(w,t\right)=\sum_{k=1}^{3}dV_{k}\left(t\right), & \; & t\in R_{+},\;M\mathrm{-a}.s. \end{array}$$

Thanks to that the uncertain process (Liu process, more precisely) w(t) is a state trajectory of DUCNNs (46), again we apply Lemma 1 (the chain rule of differentiation for Liu processes) to the uncertain process $V_{1}\left(t\right)$, given explicitly by (50), to obtain

(55) $$\begin{array}{cc} dV_{1}\left(t\right)= & 2w^{⊤}\left(t\right)\Phi dw\left(t\right)\\ = & 2w^{⊤}\left(t\right)\Phi (-Dw\left(t\right)+A_{0}{\overset{ˇ}{F}}_{0}\circ \left(w\left(t\right)\right)+A_{1}{\overset{ˇ}{F}}_{1}\circ \left(w\left(t-\tau_{1}\right)\right)\\ \; & +A_{2}\int_{t-\tau_{2}}^{t}{\overset{ˇ}{F}}_{2}\circ \left(w\left(s\right)\right)ds)dt\\ \; & +2w^{⊤}\left(t\right)\Phi (B_{0}{\overset{ˇ}{G}}_{0}\circ \left(w\left(t\right),t\right)+B_{1}{\overset{ˇ}{G}}_{1}\circ \left(w\left(t-\eta_{1}\right),t\right)\\ \; & +B_{2}\int_{t-\eta_{2}}^{t}{\overset{ˇ}{G}}_{2}\circ \left(w\left(s\right),t\right)ds)dC\left(t\right),\;t\in R_{+},\;M\mathrm{-a}.s. \end{array}$$

With the help of the experience of deriving the differential identity (55), illuminated by the definition (51) of the uncertain process $V_{2}\left(t\right)$, we have, by Lemma 1, that

(56) $$\begin{array}{cc} dV_{2}\left(t\right)= & {\left({\overset{ˇ}{F}}_{1}\circ \left(w\left(t\right)\right)\right)}^{⊤}\Psi_{1}{\overset{ˇ}{F}}_{1}\circ \left(w\left(t\right)\right)dt-{\left({\overset{ˇ}{F}}_{1}\circ \left(w\left(t-\tau_{1}\right)\right)\right)}^{⊤}\Psi_{1}{\overset{ˇ}{F}}_{1}\circ \left(w\left(t-\tau_{1}\right)\right)dt\\ \; & +w^{⊤}\left(t\right)\Psi_{2}w\left(t\right)dt-w^{⊤}\left(t-\eta_{1}\right)\Psi_{2}w\left(t-\eta_{1}\right)dt\\ \; & +w^{⊤}\left(t\right)\Psi_{3}w\left(t\right)dt-w^{⊤}\left(t-\eta_{2}\right)\Psi_{3}w\left(t-\eta_{2}\right)dt,\;t\in R_{+},\;M\mathrm{-a}.s. \end{array}$$

Enlightened by the experience gathered in the procedure of deducing the differential $dV_{1}\left(t\right)$ and $dV_{2}\left(t\right)$ (see (55) and (56) for the details) of the uncertain processes $V_{1}\left(t\right)$ and $V_{2}\left(t\right)$, by Lemma 1, we can deduce from the definition (52) of the uncertain process $V_{3}\left(t\right)$ that

(57) $$\begin{array}{cccc} \; & dV_{3}\left(t\right)=\tau_{2}w^{⊤}\left(t\right)\Omega w\left(t\right)dt-\int_{t-\tau_{2}}^{t}w^{⊤}\left(s\right)\Omega w\left(s\right)dsdt, & \; & t\in R_{+},\;M\mathrm{-a}.s. \end{array}$$

Plug the differential identities (55), (56) and (57) into the differential identity (54), and perform some routine calculations, to eventually arrive at

(58) $$\begin{array}{cc} dV(w,t)= & [2w^{⊤}\left(t\right)\Phi (-Dw\left(t\right)+A_{0}{\overset{ˇ}{F}}_{0}\circ \left(w\left(t\right)\right)+A_{1}{\overset{ˇ}{F}}_{1}\circ \left(w\left(t-\tau_{1}\right)\right)\\ \; & +A_{2}\int_{t-\tau_{2}}^{t}{\overset{ˇ}{F}}_{2}\circ \left(w\left(s\right)\right)ds)+{\left({\overset{ˇ}{F}}_{1}\circ \left(w\left(t\right)\right)\right)}^{⊤}\Psi_{1}{\overset{ˇ}{F}}_{1}\circ \left(w\left(t\right)\right)\\ \; & -{\left({\overset{ˇ}{F}}_{1}\circ \left(w\left(t-\tau_{1}\right)\right)\right)}^{⊤}\Psi_{1}{\overset{ˇ}{F}}_{1}\circ \left(w\left(t-\tau_{1}\right)\right)\\ \; & +w^{⊤}\left(t\right)\Psi_{2}w\left(t\right)-w^{⊤}\left(t-\eta_{1}\right)\Psi_{2}w\left(t-\eta_{1}\right)\\ \; & +w^{⊤}\left(t\right)\Psi_{3}w\left(t\right)-w^{⊤}\left(t-\eta_{2}\right)\Psi_{3}w\left(t-\eta_{2}\right)\\ \; & +\tau_{2}w^{⊤}\left(t\right)\Omega w\left(t\right)-\int_{t-\tau_{2}}^{t}w^{⊤}\left(s\right)\Omega w\left(s\right)ds]dt\\ \; & +2w^{⊤}\left(t\right)\Phi (B_{0}{\overset{ˇ}{G}}_{0}\circ \left(w\left(t\right),t\right)+B_{1}{\overset{ˇ}{G}}_{1}\circ \left(w\left(t-\eta_{1}\right),t\right)\\ \; & +B_{2}\int_{t-\eta_{2}}^{t}{\overset{ˇ}{G}}_{2}\circ \left(w\left(s\right),t\right)ds)dC\left(t\right),\;t\in R_{+},\;M\mathrm{-a}.s. \end{array}$$

By the fundamental theorem of uncertain calculus, we can deduce from (53) that

(59) $$\begin{array}{cc} \ln V^{\varepsilon }\left(w,t\right)= & \ln V^{\varepsilon }\left(w,0\right)+\int_{0}^{t}d\ln V^{\varepsilon }\left(w,s\right)ds=\ln V^{\varepsilon }\left(w,0\right)+\int_{0}^{t}\frac{dV^{\varepsilon }\left(w,s\right)}{V^{\varepsilon }\left(w,s\right)}\\ = & \ln V^{\varepsilon }\left(w,0\right)+\int_{0}^{t}\frac{dV\left(w,s\right)-\varepsilon e^{-\varepsilon s}ds}{V^{\varepsilon }\left(w,s\right)},\;t\in R_{+},\;M\mathrm{-a}.s. \end{array}$$

Substitute (58) into (59), and conduct some simple computations, to yield

(60) $$\begin{array}{cc} \ln V^{\varepsilon }\left(w,t\right)= & \ln V^{\varepsilon }\left(w,0\right)+\int_{0}^{t}\frac{1}{V^{\varepsilon }\left(w,s\right)}[2w^{⊤}\left(s\right)\Phi (-Dw\left(s\right)+A_{0}{\overset{ˇ}{F}}_{0}\circ \left(w\left(s\right)\right)\\ \; & +A_{1}{\overset{ˇ}{F}}_{1}\circ \left(w\left(s-\tau_{1}\right)\right)+A_{2}\int_{s-\tau_{2}}^{s}{\overset{ˇ}{F}}_{2}\circ \left(w\left(\zeta \right)\right)d\zeta )\\ \; & +{\left({\overset{ˇ}{F}}_{1}\circ \left(w\left(s\right)\right)\right)}^{⊤}\Psi_{1}{\overset{ˇ}{F}}_{1}\circ \left(w\left(s\right)\right)-{\left({\overset{ˇ}{F}}_{1}\circ \left(w\left(s-\tau_{1}\right)\right)\right)}^{⊤}\Psi_{1}{\overset{ˇ}{F}}_{1}\circ \left(w\left(s-\tau_{1}\right)\right)\\ \; & +w^{⊤}\left(s\right)\Psi_{2}w\left(s\right)-w^{⊤}\left(s-\eta_{1}\right)\Psi_{2}w\left(s-\eta_{1}\right)\\ \; & +w^{⊤}\left(s\right)\Psi_{3}w\left(s\right)-w^{⊤}\left(s-\eta_{2}\right)\Psi_{3}w\left(s-\eta_{2}\right)\\ \; & +\tau_{2}w^{⊤}\left(s\right)\Omega w\left(s\right)-\int_{t-\tau_{2}}^{t}w^{⊤}\left(s\right)\Omega w\left(s\right)ds-\varepsilon e^{-\varepsilon s}]ds\\ \; & +\int_{0}^{t}\frac{1}{V^{\varepsilon }\left(w,s\right)}2w^{⊤}\left(s\right)\Phi (B_{0}{\overset{ˇ}{G}}_{0}\circ \left(w\left(s\right),s\right)+B_{1}{\overset{ˇ}{G}}_{1}\circ \left(w\left(s-\eta_{1}\right),s\right)\\ \; & \;\;\;\;\;\;+B_{2}\int_{s-\eta_{2}}^{s}{\overset{ˇ}{G}}_{2}\circ \left(w\left(\zeta \right),s\right)d\zeta )dC\left(s\right),\;t\in R_{+},\;M\mathrm{-a}.s. \end{array}$$

To continue, our idea is to treat (60) part by part. By Lemmas 2 and 3, we have

(61) $$\begin{array}{cc} & \int_{0}^{t}\frac{2}{V^{\varepsilon }\left(w,s\right)}w^{⊤}\left(s\right)\Phi B_{0}{\overset{ˇ}{G}}_{0}\circ \left(w\left(s\right),s\right)dC\left(s\right)\\ ⩽ & k\int_{0}^{t}\frac{2}{V^{\varepsilon }\left(w,s\right)}\left|w^{⊤}\left(s\right)\Phi B_{0}{\overset{ˇ}{G}}_{0}\circ \left(w\left(s\right),s\right)\right|ds\\ ⩽ & \delta_{1}\int_{0}^{t}\frac{w^{⊤}\left(s\right)\Phi B_{0}B_{0}^{⊤}\Phi w\left(s\right)}{V^{\varepsilon }\left(w,s\right)}ds+\frac{k^{2}}{4\delta_{1}}\int_{0}^{t}\frac{{\left({\overset{ˇ}{G}}_{0}\circ \left(w\left(s\right),s\right)\right)}^{⊤}{\overset{ˇ}{G}}_{0}\circ \left(w\left(s\right),s\right)}{V^{\varepsilon }\left(w,s\right)}ds\\ ⩽ & \delta_{1}\int_{0}^{t}\frac{w^{⊤}\left(s\right)\Phi B_{0}B_{0}^{⊤}\Phi w\left(s\right)}{V^{\varepsilon }\left(w,s\right)}ds+\frac{k^{2}}{4\delta_{1}}\int_{0}^{t}\frac{w^{⊤}\left(s\right){\left(L_{G_{0}}\left(s\right)\right)}^{2}w\left(s\right)}{V^{\varepsilon }\left(w,s\right)}ds,\;t\in R_{+},\;M\mathrm{-a}.s., \end{array}$$

where the uncertain variable k is given exactly by (8) in Definition 8, the positive constant $\delta_{1}$ can be chosen as small as desired, and is therefore imagined to be very close to zero in the calculations here and hereafter. Mimic the steps in (61), to obtain

(62) $$\begin{array}{cc} \int_{0}^{t}\frac{2}{V^{\varepsilon }\left(w,s\right)}w^{⊤}\left(s\right)\Phi B_{1}{\overset{ˇ}{G}}_{1}\circ \left(w\left(s-\eta_{1}\right),s\right)dC\left(s\right)⩽ & \delta_{2}\int_{0}^{t}\frac{w^{⊤}\left(s-\eta_{1}\right)\Phi B_{1}B_{1}^{⊤}\Phi w\left(s-\eta_{1}\right)}{V^{\varepsilon }\left(w,s\right)}ds\\ \; & +\frac{k^{2}}{4\delta_{2}}\int_{0}^{t}\frac{w^{⊤}\left(s\right){\left(L_{G_{1}}\left(s\right)\right)}^{2}w\left(s\right)}{V^{\varepsilon }\left(w,s\right)}ds,\;t\in R_{+},\;M-a.s., \end{array}$$

in which Lemmas 2 and 3 played a key role, the positive constant $\delta_{2}$, as with the positive constant $\delta_{1}$ in (61), can be picked to be very close to zero (when necessary), and the uncertain variable k is defined as in Definition 8 (see (8) for the details). By performing calculations analogous to those taken in the procedure of deriving (61) as well as (62) and apply Lemmas 2 and 3, we can show finally that

(63) $$\begin{array}{cc} \; & \int_{0}^{t}\frac{2}{V^{\varepsilon }\left(w,s\right)}w^{⊤}\left(s\right)\Phi B_{2}\int_{s-\eta_{2}}^{s}{\overset{ˇ}{G}}_{2}\circ \left(w\left(\zeta \right),s\right)d\zeta dC\left(s\right)\\ ⩽ & \delta_{3}\eta_{2}\int_{0}^{t}\frac{w^{⊤}\left(s\right)\Phi B_{2}B_{2}^{⊤}\Phi w\left(s\right)}{V^{\varepsilon }\left(w,s\right)}ds\\ \; & +\frac{k^{2}}{4\delta_{3}}\int_{0}^{t}\frac{1}{V^{\varepsilon }\left(w,s\right)}\int_{s-\eta_{2}}^{s}w^{⊤}\left(\zeta \right){\left(L_{G_{2}}\left(s\right)\right)}^{2}w\left(\zeta \right)d\zeta ds,\;t\in R_{+},\;M\mathrm{-a}.s., \end{array}$$

where k, as in (61) and (62), is an uncertain variable whose definition lies in (8) of Definition 8, and the real constant $\delta_{3}$, required to be positive, can be chosen to be as close to zero as desired. Now let us plug (61), (62) and (63) into (60), to arrive at

(64) $$\begin{array}{cc} \ln V^{\varepsilon }\left(w,t\right)⩽ & \ln V^{\varepsilon }\left(w,0\right)+\int_{0}^{t}\frac{1}{V^{\varepsilon }\left(w,s\right)}[2w^{⊤}\left(s\right)\Phi (-Dw\left(s\right)+A_{0}{\overset{ˇ}{F}}_{0}\circ \left(w\left(s\right)\right)\\ \; & +A_{1}{\overset{ˇ}{F}}_{1}\circ \left(w\left(s-\tau_{1}\right)\right)+A_{2}\int_{s-\tau_{2}}^{s}{\overset{ˇ}{F}}_{2}\circ \left(w\left(\zeta \right)\right)d\zeta )\\ \; & +{\left({\overset{ˇ}{F}}_{1}\circ \left(w\left(s\right)\right)\right)}^{⊤}\Psi_{1}{\overset{ˇ}{F}}_{1}\circ \left(w\left(s\right)\right)\\ \; & -{\left({\overset{ˇ}{F}}_{1}\circ \left(w\left(s-\tau_{1}\right)\right)\right)}^{⊤}\Psi_{1}{\overset{ˇ}{F}}_{1}\circ \left(w\left(s-\tau_{1}\right)\right)\\ \; & +w^{⊤}\left(s\right)\Psi_{2}w\left(s\right)-w^{⊤}\left(s-\eta_{1}\right)\Psi_{2}w\left(s-\eta_{1}\right)\\ \; & +w^{⊤}\left(s\right)\Psi_{3}w\left(s\right)-w^{⊤}\left(s-\eta_{2}\right)\Psi_{3}w\left(s-\eta_{2}\right)\\ \; & +\tau_{2}w^{⊤}\left(s\right)\Omega w\left(s\right)-\int_{t-\tau_{2}}^{t}w^{⊤}\left(s\right)\Omega w\left(s\right)ds-\varepsilon e^{-\varepsilon s}]ds\\ \; & +\delta_{1}\int_{0}^{t}\frac{w^{⊤}\left(s\right)\Phi B_{0}B_{0}^{⊤}\Phi w\left(s\right)}{V^{\varepsilon }\left(w,s\right)}ds+\frac{k^{2}}{4\delta_{1}}\int_{0}^{t}\frac{w^{⊤}\left(s\right){\left(L_{G_{0}}\left(s\right)\right)}^{2}w\left(s\right)}{V^{\varepsilon }\left(w,s\right)}ds\\ \; & +\delta_{2}\int_{0}^{t}\frac{w^{⊤}\left(s-\eta_{1}\right)\Phi B_{1}B_{1}^{⊤}\Phi w\left(s-\eta_{1}\right)}{V^{\varepsilon }\left(w,s\right)}ds\\ \; & +\frac{k^{2}}{4\delta_{2}}\int_{0}^{t}\frac{w^{⊤}\left(s\right){\left(L_{G_{1}}\left(s\right)\right)}^{2}w\left(s\right)}{V^{\varepsilon }\left(w,s\right)}ds+\delta_{3}\eta_{2}\int_{0}^{t}\frac{w^{⊤}\left(s\right)\Phi B_{2}B_{2}^{⊤}\Phi w\left(s\right)}{V^{\varepsilon }\left(w,s\right)}ds\\ \; & +\frac{k^{2}}{4\delta_{3}}\int_{0}^{t}\frac{1}{V^{\varepsilon }\left(w,s\right)}\int_{s-\eta_{2}}^{s}w^{⊤}\left(\zeta \right){\left(L_{G_{2}}\left(s\right)\right)}^{2}w\left(\zeta \right)d\zeta ds,\;t\in R_{+},\;M\mathrm{-a}.s., \end{array}$$

in which, the occurred diagonal matrix $L_{G_{k}}\left(s\right)$ is defined as in (36) alongside with (37) in Assumption 3, k=0, 1, 2. By recalling that the nontrivial entries of the diagonal matrix $L_{G_{k}}\left(s\right)$ are Lebesgue integrable and essentially bounded, k=0, 1, 2, and in view of

$$\begin{array}{cccc} \; & V^{\varepsilon }\left(w,s\right)=V^{\varepsilon }\left(w,s\right)+e^{-\varepsilon s}⩾e^{-\varepsilon s}, & \; & s\in R_{+}, \end{array}$$

we can conclude immediately that the terms (occurred in (64), (61), (62) as well as (63))

$$\begin{array}{c} \frac{k^{2}}{4\delta_{1}}\int_{0}^{t}\frac{w^{⊤}\left(s\right){\left(L_{G_{0}}\left(s\right)\right)}^{2}w\left(s\right)}{V^{\varepsilon }\left(w,s\right)}ds,\;\frac{k^{2}}{4\delta_{2}}\int_{0}^{t}\frac{w^{⊤}\left(s\right){\left(L_{G_{1}}\left(s\right)\right)}^{2}w\left(s\right)}{V^{\varepsilon }\left(w,s\right)}ds,\;\mathrm{and}\\ \frac{k^{2}}{4\delta_{3}}\int_{0}^{t}\frac{1}{V^{\varepsilon }\left(w,s\right)}\int_{s-\eta_{2}}^{s}w^{⊤}\left(\zeta \right){\left(L_{G_{2}}\left(s\right)\right)}^{2}w\left(\zeta \right)d\zeta ds \end{array}$$

are well-defined as uncertain processes (Liu process, more precisely). Based on (33) along with (34) in Assumption 3, and by the famous Cauchy–Schwarz inequality, we have

(65) $$\begin{array}{cc} 2w^{⊤}\left(s\right)\Phi A_{0}{\overset{ˇ}{F}}_{0}\circ \left(w\left(s\right)\right)⩽ & w^{⊤}\left(s\right)\Phi A_{0}A_{0}^{⊤}\Phi^{⊤}w\left(s\right)+w^{⊤}\left(s\right){\left(L_{F_{0}}\right)}^{2}w\left(s\right)\\ = & w^{⊤}\left(s\right)\left(\Phi A_{0}A_{0}^{⊤}\Phi^{⊤}+{\left(L_{F_{0}}\right)}^{2}\right)w\left(s\right),\;s\in R_{+},\;M\mathrm{-a}.s. \end{array}$$

By Lemmas 5 (especially (22)) and 4 ((18), in particular), we have directly

(66) $$\begin{array}{cccc} & {\left({\overset{ˇ}{F}}_{1}\circ \left(w\left(s\right)\right)\right)}^{⊤}\Psi_{1}{\overset{ˇ}{F}}_{1}\circ \left(w\left(s\right)\right)⩽\lambda_{\max}\left(\Psi_{1}\right)w^{⊤}\left(s\right)L_{F_{1}}w\left(s\right),\; & \; & t\in R_{+},\;M\mathrm{-a}.s. \end{array}$$

Based on the idea used in (66), with the Cauchy–Schwarz inequality as the main tool, we apply Lemmas 5 and 4 ((22) and (18), in particular), to obtain

(67) $$\begin{array}{cccc} \; & \tau_{2}{\left({\overset{ˇ}{F}}_{2}\circ \left(w\left(s\right)\right)\right)}^{⊤}\Omega {\overset{ˇ}{F}}_{2}\circ \left(w\left(s\right)\right)⩽\tau_{2}\lambda_{\max}\left(\Omega \right)w^{⊤}\left(s\right)L_{F_{2}}w\left(s\right),\; & \; & t\in R_{+},\;M\mathrm{-a}.s. \end{array}$$

As with $L_{F_{0}}$ in (65), the diagonal matrices $L_{F_{1}}$ and $L_{F_{2}}$, occurred in (66) and (67), are defined as in (33) alongside with (34) in Assumption 3. Based on (32) in Assumption 3, the right hand sides of (65), (66) and (67) are all well-defined.

With (48), (49) as well as (50) at our disposal, we perform some routine but seemingly tedious calculations, to arrive at

(68) $$\begin{array}{cc} \frac{w^{⊤}\left(s\right){\left(L_{G_{0}}\left(s\right)\right)}^{2}w\left(s\right)}{V^{\varepsilon }\left(w,s\right)}⩽ & \frac{\mathrm{tr}\left({\left(L_{G_{0}}\left(s\right)\right)}^{2}\right)w^{⊤}\left(s\right)w\left(s\right)}{V^{\varepsilon }\left(w,s\right)}\\ ⩽ & \frac{\mathrm{tr}\left({\left(L_{G_{0}}\left(s\right)\right)}^{2}\right)w^{⊤}\left(s\right)\Phi w\left(s\right)}{(\min_{1⩽k⩽N}\varphi_{k})V^{\varepsilon }\left(w,s\right)}\\ ⩽ & \frac{\mathrm{tr}\left({\left(L_{G_{0}}\left(s\right)\right)}^{2}\right)}{\min_{1⩽k⩽N}\varphi_{k}}\;\mathrm{for}\;a.e.\;s\in R_{+},\;M\mathrm{-a}.s. \end{array}$$

This implies automatically

(69) $$\begin{array}{cccc} \int_{0}^{t}\frac{w^{⊤}\left(s\right){\left(L_{G_{0}}\left(s\right)\right)}^{2}w\left(s\right)}{V^{\varepsilon }\left(w,s\right)}ds⩽ & \frac{1}{\min_{1⩽k⩽N}\varphi_{k}}\int_{0}^{t}\mathrm{tr}\left({\left(L_{G_{0}}\left(s\right)\right)}^{2}\right)ds & \; & \\ ⩽ & \frac{1}{\min_{1⩽k⩽N}\varphi_{k}}\int_{0}^{+\infty }\mathrm{tr}\left({\left(L_{G_{0}}\left(s\right)\right)}^{2}\right)ds,\; & \; & t\in R_{+},\;M\mathrm{-a}.s. \end{array}$$

Borrowing the idea ‘to establish first the inequality (68) and based on this new established inequality (68), to prove our desired (69)’, based on (48), (49) and (50), we have analogously

(70) $$\begin{array}{cccc} \int_{0}^{t}\frac{w^{⊤}\left(s\right){\left(L_{G_{1}}\left(s\right)\right)}^{2}w\left(s\right)}{V^{\varepsilon }\left(w,s\right)}ds⩽ & \frac{1}{\min_{1⩽k⩽N}\varphi_{k}}\int_{0}^{t}\mathrm{tr}\left({\left(L_{G_{1}}\left(s\right)\right)}^{2}\right)ds & \; & \\ ⩽ & \frac{1}{\min_{1⩽k⩽N}\varphi_{k}}\int_{0}^{+\infty }\mathrm{tr}\left({\left(L_{G_{1}}\left(s\right)\right)}^{2}\right)ds,\; & \; & t\in R_{+},\;M\mathrm{-a}.s. \end{array}$$

Enlightened by the experience of deducing (68), based on (48), (49) and (51), we conduct some careful computations, to yield

$$\begin{array}{cc} \frac{1}{V^{\varepsilon }\left(w,s\right)}\int_{s-\eta_{2}}^{s}w^{⊤}\left(\zeta \right){\left(L_{G_{2}}\left(s\right)\right)}^{2}w\left(\zeta \right)d\zeta ⩽ & \frac{\mathrm{tr}\left({\left(L_{G_{2}}\left(s\right)\right)}^{2}\right)}{\lambda_{\min}\left(\Psi_{3}\right)V^{\varepsilon }\left(w,s\right)}\int_{s-\eta_{2}}^{s}\lambda_{\min}\left(\Psi_{3}\right)w^{⊤}\left(\zeta \right)w\left(\zeta \right)d\zeta \\ ⩽ & \frac{\mathrm{tr}\left({\left(L_{G_{2}}\left(s\right)\right)}^{2}\right)}{\lambda_{\min}\left(\Psi_{3}\right)V^{\varepsilon }\left(w,s\right)}\int_{s-\eta_{2}}^{s}w^{⊤}\left(\zeta \right)\Psi_{3}w\left(\zeta \right)d\zeta \\ ⩽ & \frac{\mathrm{tr}\left({\left(L_{G_{2}}\left(s\right)\right)}^{2}\right)}{\lambda_{\min}\left(\Psi_{3}\right)}\;\mathrm{for}\;a.e.\;s\in R_{+},\;M\mathrm{-a}.s. \end{array}$$

As can be seen already in (69), this implies directly

(71) $$\begin{array}{cccc} \int_{0}^{t}\frac{1}{V^{\varepsilon }\left(w,s\right)}\int_{s-\eta_{2}}^{s}w^{⊤}\left(\zeta \right){\left(L_{G_{2}}\left(s\right)\right)}^{2}w\left(\zeta \right)d\zeta ds⩽ & \frac{1}{\lambda_{\min}\left(\Psi_{3}\right)}\int_{0}^{t}\mathrm{tr}\left({\left(L_{G_{2}}\left(s\right)\right)}^{2}\right)ds & \; & \\ ⩽ & \frac{1}{\lambda_{\min}\left(\Psi_{3}\right)}\int_{0}^{+\infty }\mathrm{tr}\left({\left(L_{G_{2}}\left(s\right)\right)}^{2}\right)ds,\; & \; & t\in R_{+},\;M\mathrm{-a}.s. \end{array}$$

By recalling (35) in Assumption 3, we conclude that the terms in (69), (70) and (71)

$$\begin{array}{c} \frac{1}{\min_{1⩽k⩽N}\varphi_{k}}\int_{0}^{+\infty }\mathrm{tr}\left({\left(L_{G_{0}}\left(s\right)\right)}^{2}\right)ds,\;\frac{1}{\min_{1⩽k⩽N}\varphi_{k}}\int_{0}^{+\infty }\mathrm{tr}\left({\left(L_{G_{1}}\left(s\right)\right)}^{2}\right)ds,\;\mathrm{and}\\ \frac{1}{\lambda_{\min}\left(\Psi_{3}\right)}\int_{0}^{+\infty }\mathrm{tr}\left({\left(L_{G_{2}}\left(s\right)\right)}^{2}\right)ds \end{array}$$

are all well-defined as non-negative constants.

Plug (69), (70) and (71) into (64), to directly obtain

$$\begin{array}{cc} \ln V^{\varepsilon }\left(w,t\right)⩽ & \ln V^{\varepsilon }\left(w,0\right)+\int_{0}^{t}\frac{1}{V^{\varepsilon }\left(w,s\right)}[-2w^{⊤}\left(s\right)\Phi Dw\left(s\right)\\ \; & +w^{⊤}\left(s\right)\left(\Phi A_{0}A_{0}^{⊤}\Phi^{⊤}+{\left(L_{F_{0}}\right)}^{2}\right)w\left(s\right)\\ \; & +2w^{⊤}\left(s\right)\Phi A_{1}{\overset{ˇ}{F}}_{1}\circ \left(w\left(s-\tau_{1}\right)\right)+2w^{⊤}\left(s\right)\Phi A_{2}\int_{s-\tau_{2}}^{s}{\overset{ˇ}{F}}_{2}\circ \left(w\left(\zeta \right)\right)d\zeta \\ \; & +\lambda_{\max}\left(\Psi_{1}\right)w^{⊤}\left(s\right)L_{F_{1}}w\left(s\right)\\ \; & -{\left({\overset{ˇ}{F}}_{1}\circ \left(w\left(s-\tau_{1}\right)\right)\right)}^{⊤}\Psi_{1}{\overset{ˇ}{F}}_{1}\circ \left(w\left(s-\tau_{1}\right)\right)\\ \; & +w^{⊤}\left(s\right)\Psi_{2}w\left(s\right)-w^{⊤}\left(s-\eta_{1}\right)\Psi_{2}w\left(s-\eta_{1}\right)\\ \; & +w^{⊤}\left(s\right)\Psi_{3}w\left(s\right)-w^{⊤}\left(s-\eta_{2}\right)\Psi_{3}w\left(s-\eta_{2}\right)\\ \; & +\tau_{2}w^{⊤}\left(s\right)\Omega w\left(s\right)-\int_{t-\tau_{2}}^{t}w^{⊤}\left(s\right)\Omega w\left(s\right)ds-\varepsilon e^{-\varepsilon s}]ds\\ \; & +\delta_{1}\int_{0}^{t}\frac{w^{⊤}\left(s\right)\Phi B_{0}B_{0}^{⊤}\Phi w\left(s\right)}{V^{\varepsilon }\left(w,s\right)}ds+\frac{k^{2}}{4\delta_{1}\min_{1⩽k⩽N}\varphi_{k}}\int_{0}^{+\infty }\mathrm{tr}\left({\left(L_{G_{0}}\left(s\right)\right)}^{2}\right)ds\\ \; & +\delta_{2}\int_{0}^{t}\frac{w^{⊤}\left(s-\eta_{1}\right)\Phi B_{1}B_{1}^{⊤}\Phi w\left(s-\eta_{1}\right)}{V^{\varepsilon }\left(w,s\right)}ds\\ \; & +\frac{k^{2}}{4\delta_{2}\min_{1⩽k⩽N}\varphi_{k}}\int_{0}^{+\infty }\mathrm{tr}\left({\left(L_{G_{1}}\left(s\right)\right)}^{2}\right)ds+\delta_{3}\eta_{2}\int_{0}^{t}\frac{w^{⊤}\left(s\right)\Phi B_{2}B_{2}^{⊤}\Phi w\left(s\right)}{V^{\varepsilon }\left(w,s\right)}ds\\ \; & +\frac{k^{2}}{4\delta_{3}\lambda_{\min}\left(\Psi_{3}\right)}\int_{0}^{+\infty }\mathrm{tr}\left({\left(L_{G_{2}}\left(s\right)\right)}^{2}\right)ds,\;t\in R_{+},\;M\mathrm{-a}.s. \end{array}$$

which can be written compactly into

(72) $$\begin{array}{cc} \frac{\ln V^{\varepsilon }\left(w,t\right)}{t}⩽ & \frac{\ln V^{\varepsilon }\left(w,0\right)}{t}-\int_{0}^{t}\frac{1}{V^{\varepsilon }\left(w,s\right)}[{\tilde{w}}^{⊤}\left(s\right)\Theta \tilde{w}\left(s\right)\\ \; & +w^{⊤}\left(s-\eta_{1}\right)\left(\Psi_{2}-\delta_{2}\Phi B_{1}B_{1}^{⊤}\Phi \right)w\left(s-\eta_{1}\right)\\ \; & +w^{⊤}\left(s-\eta_{2}\right)\Psi_{3}w\left(s-\eta_{2}\right)\\ \; & +\int_{s-\tau_{2}}^{s}w^{⊤}\left(\zeta \right)\Omega w\left(\zeta \right)d\zeta +\varepsilon e^{-\varepsilon s}]ds\\ \; & +\frac{k^{2}}{4\delta_{1}t\min_{1⩽k⩽N}\varphi_{k}}\int_{0}^{+\infty }\mathrm{tr}\left({\left(L_{G_{0}}\left(s\right)\right)}^{2}\right)ds\\ \; & +\frac{k^{2}}{4\delta_{2}t\min_{1⩽k⩽N}\varphi_{k}}\int_{0}^{+\infty }\mathrm{tr}\left({\left(L_{G_{1}}\left(s\right)\right)}^{2}\right)ds\\ \; & +\frac{k^{2}}{4\delta_{3}t\lambda_{\min}\left(\Psi_{3}\right)}\int_{0}^{+\infty }\mathrm{tr}\left({\left(L_{G_{2}}\left(s\right)\right)}^{2}\right)ds,\;t\in R_{+},\;M\mathrm{-a}.s., \end{array}$$

in which the uncertain process $\tilde{w}\left(s\right)$ is defined by

(73) $$\begin{array}{cccc} \; & \tilde{w}\left(s\right)=\mathrm{col}(w\left(s\right),\;{\overset{ˇ}{F}}_{1}\circ \left(w\left(s-\tau_{1}\right)\right),\;\int_{s-\tau_{2}}^{s}{\overset{ˇ}{F}}_{2}\circ \left(w\left(\zeta \right)\right)d\zeta ),\; & \; & s\in R_{+},\;M\mathrm{-a}.s., \end{array}$$

and the symmetric block matrix Θ is defined as in (42). Since the block matrix Θ is positive definite, it follows immediately from Lemma 4 that

(74) $$\begin{array}{cccc} V_{1}\left(s\right)= & w^{⊤}\left(s\right)\Phi w\left(s\right) & \; & \\ = & {\tilde{w}}^{⊤}\left(s\right)\tilde{\Phi }\tilde{w}\left(s\right) & \; & \\ ⩽ & \lambda_{\max}\left({\left({⨿}_{\Theta }^{-1}\right)}^{⊤}\tilde{\Phi }{⨿}_{\Theta }^{-1}\right){\tilde{w}}^{⊤}\left(s\right)\Theta \tilde{w}\left(s\right), & \; & s\in R_{+},\;M\mathrm{-a}.s., \end{array}$$

where $\tilde{w}\left(s\right)$ is given as in (73) and the symmetric block matrix $\tilde{\Phi }$ is given by (44). Since the matrices $\Psi_{1}$, $\Psi_{2}$ and $\Psi_{3}$ are all positive definite, $\tau_{2}⩾\tau_{1}$, $\tau_{2}⩾\eta_{1}$ and $\tau_{2}⩾\eta_{2}$ (see Assumption 2), it follows from the Cauchy–Schwarz inequality, Lemmas 5 and 4 that

(75) $$\begin{array}{cc} V_{2}\left(s\right)= & \int_{s-\tau_{1}}^{s}{\left({\overset{ˇ}{F}}_{1}\circ \left(w\left(\zeta \right)\right)\right)}^{⊤}\Psi_{1}{\overset{ˇ}{F}}_{1}\circ \left(w\left(\zeta \right)\right)d\zeta +\int_{s-\eta_{1}}^{s}w^{⊤}\left(\zeta \right)\Psi_{2}w\left(\zeta \right)ds\\ \; & +\int_{s-\eta_{2}}^{s}w^{⊤}\left(\zeta \right)\Psi_{3}w\left(\zeta \right)d\zeta \\ ⩽ & \lambda_{\max}\left(\Psi_{1}\right)\int_{s-\tau_{2}}^{s}w^{⊤}\left(\zeta \right){\left(L_{F_{1}}\right)}^{2}w\left(\zeta \right)d\zeta +\int_{s-\tau_{2}}^{s}w^{⊤}\left(\zeta \right)\Psi_{2}w\left(\zeta \right)d\zeta \\ \; & +\int_{s-\tau_{2}}^{s}w^{⊤}\left(\zeta \right)\Psi_{3}w\left(\zeta \right)d\zeta \\ ⩽ & (\lambda_{\max}\left(\Psi_{1}\right)\lambda_{\max}\left({\left({⨿}_{\Omega }^{-1}\right)}^{⊤}{\left(L_{F_{1}}\right)}^{2}{⨿}_{\Omega }^{-1}\right)+\lambda_{\max}\left({\left({⨿}_{\Omega }^{-1}\right)}^{⊤}\Psi_{2}{⨿}_{\Omega }^{-1}\right)\\ \; & +\lambda_{\max}\left({\left({⨿}_{\Omega }^{-1}\right)}^{⊤}\Psi_{3}{⨿}_{\Omega }^{-1}\right))\int_{s-\tau_{2}}^{s}w^{⊤}\left(\zeta \right)\Omega w\left(\zeta \right)d\zeta ,\;s\in R_{+},\;M\mathrm{-a}.s. \end{array}$$

It is not difficult to find that

(76) $$\begin{array}{cc} V_{3}\left(s\right)= & \int_{s-\tau_{2}}^{s}\int_{\varrho }^{s}w^{⊤}\left(\zeta \right)\Omega w\left(\zeta \right)d\zeta d\varrho \\ ⩽ & \tau_{2}\int_{s-\tau_{2}}^{s}w^{⊤}\left(\zeta \right)\Omega w\left(\zeta \right)d\zeta ,\;s\in R_{+},\;M\mathrm{-a}.s. \end{array}$$

Plug (74), (75) and (76) into (49), to obtain

$$\begin{array}{cc} V(w,s)⩽ & \sum_{k=1}^{3}V_{k}\left(s\right)\\ ⩽ & \lambda_{\max}\left({\left({⨿}_{\Theta }^{-1}\right)}^{⊤}\tilde{\Phi }{⨿}_{\Theta }^{-1}\right){\tilde{w}}^{⊤}\left(s\right)\Theta \tilde{w}\left(s\right)\\ \; & +(\lambda_{\max}\left(\Psi_{1}\right)\lambda_{\max}\left({\left({⨿}_{\Omega }^{-1}\right)}^{⊤}{\left(L_{F_{1}}\right)}^{2}{⨿}_{\Omega }^{-1}\right)+\lambda_{\max}\left({\left({⨿}_{\Omega }^{-1}\right)}^{⊤}\Psi_{2}{⨿}_{\Omega }^{-1}\right)\\ \; & +\lambda_{\max}\left({\left({⨿}_{\Omega }^{-1}\right)}^{⊤}\Psi_{3}{⨿}_{\Omega }^{-1}\right)+\tau_{2})\int_{s-\tau_{2}}^{s}w^{⊤}\left(\zeta \right)\Omega w\left(\zeta \right)d\zeta ,\;s\in R_{+},\;M\mathrm{-a}.s. \end{array}$$

This implies immediately that

(77) $$\begin{array}{cccc} \; & {\tilde{w}}^{⊤}\left(s\right)\Theta \tilde{w}\left(s\right)+\int_{s-\tau_{2}}^{s}w^{⊤}\left(\zeta \right)\Omega w\left(\zeta \right)d\zeta ⩾\kappa V\left(w,s\right), & \; & s\in R_{+},\;M\mathrm{-a}.s., \end{array}$$

where the positive constant κ is given such that (43) is fulfilled.

Thanks to the assumption that the matrices $\Psi_{2}-\delta_{2}\Phi B_{1}B_{1}^{⊤}\Phi$ and $\Psi_{3}$ are both positive definite, we plug (77) into (72) to arrive at

$$\begin{array}{cc} \frac{\ln V^{\varepsilon }\left(w,t\right)}{t}⩽ & \frac{\ln V^{\varepsilon }\left(w,0\right)}{t}-\int_{0}^{t}\frac{\kappa V\left(w,s\right)+\varepsilon e^{-\varepsilon s}}{V\left(w,s\right)+e^{-\varepsilon s}}ds\\ \; & +\frac{k^{2}}{4\delta_{1}t\min_{1⩽k⩽N}\varphi_{k}}\int_{0}^{+\infty }\mathrm{tr}\left({\left(L_{G_{0}}\left(s\right)\right)}^{2}\right)ds\\ \; & +\frac{k^{2}}{4\delta_{2}t\min_{1⩽k⩽N}\varphi_{k}}\int_{0}^{+\infty }\mathrm{tr}\left({\left(L_{G_{1}}\left(s\right)\right)}^{2}\right)ds\\ \; & +\frac{k^{2}}{4\delta_{3}t\lambda_{\min}\left(\Psi_{3}\right)}\int_{0}^{+\infty }\mathrm{tr}\left({\left(L_{G_{2}}\left(s\right)\right)}^{2}\right)ds,\;t\in R_{+},\;M\mathrm{-a}.s. \end{array}$$

Fix ε=κ, and pass to the limit as t→+∞ to finally obtain that

$$\begin{array}{cccc} \; & \underset{t\to +\infty }{lim sup}\frac{\ln V^{\varepsilon }\left(w,t\right)}{t}⩽-\kappa , & \; & M\mathrm{-a}.s. \end{array}$$

For every state trajectory x(t) of DUCNNs (23), if $x\left(t\right)\neq x^{*}$ (recall that $x^{*}$ is an equilibrium state or a fixed point of DUCNNs (23)) for every $t\in R_{+}$, then it holds that

(78) $$\begin{array}{cc} \underset{t\to +\infty }{lim sup}\frac{\ln\left({\left(x\left(t\right)-x^{*}\right)}^{⊤}\Phi \left(x\left(t\right)-x^{*}\right)\right)}{t}⩽ & \underset{t\to +\infty }{lim sup}\frac{\ln V(w,t)}{t}\\ ⩽ & \underset{t\to +\infty }{lim sup}\frac{\ln V^{\varepsilon }\left(w,t\right)}{t}⩽-\kappa ,\;M\mathrm{-a}.s. \end{array}$$

The proof of Theorem 2 is complete. □

## 4. Numerical Validation of the Theoretical Observations

In Section 3, we provided a criterion ensuring the (unique) existence of the equilibrium state (or fixed point) of DUCNNs (23) and proved a criterion guaranteeing the convergence of state trajectories of our concerned NNs. In this section, we are focused in coming up with an example to illustrate that the aforementioned theoretical results are indeed effective.

We consider a DUCNN having the form (23) with N=3, $x={\left(x_{1},x_{2},x_{3}\right)}^{⊤}$. We assume that the delay $\tau_{1}$, $\tau_{2}$, $\eta_{1}$ and $\eta_{2}$ are given by $\tau_{1}=1$, $\tau_{2}=4$, $\eta_{1}=2$ and $\eta_{2}=3$, respectively. We assume in our concerned example that the matrix in the leakage term is

$$D=\left(\begin{array}{ccc} 500 & 0 & 0\\ 0 & 500 & 0\\ 0 & 0 & 500 \end{array}\right),$$

and that the connection weight coefficient matrices $A_{0}$, $A_{1}$, $A_{2}$, $B_{0}$, $B_{1}$ and $B_{2}$ of the transmission terms are given, respectively, by

$$\begin{array}{cccccc} \; & A_{0}=\left(\begin{array}{ccc} 3 & 1 & 9\\ 11 & 12 & 3\\ 6 & 2 & 7 \end{array}\right),\; & \; & A_{1}=\left(\begin{array}{ccc} 8 & 9 & 3\\ 7 & 4 & 12\\ 11 & 10 & 2 \end{array}\right),\; & \; & A_{2}=\left(\begin{array}{ccc} 1 & 11 & 13\\ 8 & 10 & 2\\ 6 & 12 & 5 \end{array}\right),\\ \; & B_{0}=\left(\begin{array}{ccc} 10 & 11 & 1\\ 7 & 6 & 13\\ 5 & 12 & 3 \end{array}\right),\; & \; & B_{1}=\left(\begin{array}{ccc} 11 & 9 & 3\\ 7 & 4 & 13\\ 8 & 10 & 2 \end{array}\right),\; & \; & \mathrm{and}\;B_{2}=\left(\begin{array}{ccc} 3 & 11 & 7\\ 8 & 10 & 2\\ 6 & 12 & 5 \end{array}\right). \end{array}$$

For the sake of convenience of our later computations, we assume in this example that the exogenous disturbance U(t) and V(t) are zero for all $t\in R_{+}$.

We assume in our concerned example DUCNN that the activation functions $F_{0}$, $F_{1}$, $F_{2}$, $G_{0}$, $G_{1}$ and $G_{2}$ are given, respectively, by

$$\begin{array}{cccc} F_{0}\circ \left(x\right)= & {\left(\frac{|x_{1}+1|-|x_{1}-1|}{2},\frac{|2x_{2}+1|-|2x_{2}-1|}{2},\frac{|3x_{3}+1|-|3x_{3}-1|}{2}\right)}^{⊤}, & \; & x={\left(x_{1},x_{2},x_{3}\right)}^{⊤}\in R^{3}, \end{array}$$

$$\begin{array}{cccc} F_{1}\circ \left(x\right)= & {\left(\frac{|2x_{1}+1|-|2x_{1}-1|}{2},\frac{|x_{2}+1|-|x_{2}-1|}{2},\frac{|3x_{3}+1|-|3x_{3}-1|}{2}\right)}^{⊤}, & \; & x={\left(x_{1},x_{2},x_{3}\right)}^{⊤}\in R^{3}, \end{array}$$

$$\begin{array}{cccc} F_{2}\circ \left(x\right)= & {\left(\frac{|3x_{1}+1|-|3x_{1}-1|}{2},\frac{|2x_{2}+1|-|2x_{2}-1|}{2},\frac{|x_{3}+1|-|x_{3}-1|}{2}\right)}^{⊤}, & \; & x={\left(x_{1},x_{2},x_{3}\right)}^{⊤}\in R^{3}, \end{array}$$

$$\begin{array}{cccc} G_{0}\circ \left(x,t\right)= & {\left(\frac{|x_{1}+1|-|x_{1}-1|}{2(1+t)},\frac{|3x_{2}+1|-|3x_{2}-1|}{2(1+t)},\frac{|2x_{3}+1|-|2x_{3}-1|}{2(1+t)}\right)}^{⊤}, & \; & x={\left(x_{1},x_{2},x_{3}\right)}^{⊤}\in R^{3},\;t\in R_{+}, \end{array}$$

$$\begin{array}{cccc} G_{1}\circ \left(x,t\right)= & {\left(\frac{|2x_{1}+1|-|2x_{1}-1|}{2(1+t)},\frac{|3x_{2}+1|-|3x_{2}-1|}{2(1+t)},\frac{|x_{3}+1|-|x_{3}-1|}{2(1+t)}\right)}^{⊤}, & \; & x={\left(x_{1},x_{2},x_{3}\right)}^{⊤}\in R^{3},\;t\in R_{+} \end{array}$$

and

$$\begin{array}{cccc} G_{2}\circ \left(x,t\right)= & {\left(\frac{|3x_{1}+1|-|3x_{1}-1|}{2(1+t)},\frac{|x_{2}+1|-|x_{2}-1|}{2(1+t)},\frac{|2x_{3}+1|-|2x_{3}-1|}{2(1+t)}\right)}^{⊤}, & \; & x={\left(x_{1},x_{2},x_{3}\right)}^{⊤}\in R^{3},\;t\in R_{+}. \end{array}$$

With the above given $\tau_{1}$, $\tau_{2}$, $\eta_{1}$, $\eta_{2}$, $A_{0}$, $A_{1}$, $A_{2}$, $B_{0}$, $B_{1}$, $B_{2}$, Φ, $\Psi_{1}$, $F_{0}$, $F_{1}$, $F_{2}$, $G_{0}$, $G_{1}$ and $G_{2}$, we can prove easily that our concerned example DUCNN admits $x={\left(0,0,0\right)}^{⊤}$ as its equilibrium state (or fixed point). Next, we would like to check numerically and graphically that $x={\left(0,0,0\right)}^{⊤}$ is actually the unique equilibrium state (or fixed point) of our concerned example DUCNN, moreover, it is almost surely exponentially stable. By some routine but seemingly tedious calculations, we have

$$\begin{array}{cccccc} \; & L_{F_{0}}=\left(\begin{array}{ccc} 1 & 0 & 0\\ 0 & 2 & 0\\ 0 & 0 & 3 \end{array}\right),\; & \; & L_{F_{1}}=\left(\begin{array}{ccc} 2 & 0 & 0\\ 0 & 1 & 0\\ 0 & 0 & 3 \end{array}\right),\; & \; & L_{F_{2}}=\left(\begin{array}{ccc} 3 & 0 & 0\\ 0 & 2 & 0\\ 0 & 0 & 1 \end{array}\right),\\ \; & L_{G_{0}}=\frac{1}{1+t}\left(\begin{array}{ccc} 1 & 0 & 0\\ 0 & 3 & 0\\ 0 & 0 & 2 \end{array}\right),\; & \; & L_{G_{1}}=\frac{1}{1+t}\left(\begin{array}{ccc} 2 & 0 & 0\\ 0 & 3 & 0\\ 0 & 0 & 1 \end{array}\right),\; & \; & L_{G_{2}}=\frac{1}{1+t}\left(\begin{array}{ccc} 3 & 0 & 0\\ 0 & 1 & 0\\ 0 & 0 & 2 \end{array}\right). \end{array}$$

To reduce the computational burden, we choose to fix Φ and $\Psi_{1}$ as

(79) $$\Phi =\Psi_{1}=\left(\begin{array}{ccc} 1 & 0 & 0\\ 0 & 1 & 0\\ 0 & 0 & 1 \end{array}\right).$$

We determine $\Psi_{2}$, $\Psi_{3}$ and Ω by solving linear matrix inequalities (LMIs) (41) and (42) (with merely $\Psi_{2}$, $\Psi_{3}$ and Ω as the decision variables) via exploiting MATLAB (^®^2015b), to obtain

$$\Psi_{2}=\left(\begin{array}{ccc} 244.8509 & -73.0298 & -94.5331\\ -73.0298 & 161.1998 & -91.6697\\ -94.5331 & -91.6697 & 218.0977 \end{array}\right),$$

$$\Psi_{3}=\left(\begin{array}{ccc} 118.1714 & -39.9782 & -50.9662\\ -39.9782 & 76.1573 & -48.7077\\ -50.9662 & -48.7077 & 105.7461 \end{array}\right),$$

and

$$\Omega =\left(\begin{array}{ccc} 60.5751 & -16.9317 & -24.0696\\ -16.9317 & 44.6221 & -20.9097\\ -24.0696 & -20.9097 & 54.8402 \end{array}\right).$$

With the above fixed $\tau_{1}$, $\tau_{2}$, $\eta_{1}$, $\eta_{2}$, $A_{0}$, $A_{1}$, $A_{2}$, $B_{0}$, $B_{1}$, $B_{2}$, Φ, $\Psi_{1}$, and with the obtained $L_{F_{0}}$, $L_{F_{1}}$, $L_{F_{2}}$, $L_{G_{0}}$, $L_{G_{1}}$, $L_{G_{2}}$, $\Psi_{2}$, $\Psi_{3}$ and Ω, we again perform some numerical computations via MATLAB (^®^2015b) and obtain ς=0.8261 and κ=0.0947; see (38) and (43) for the detailed definitions of ς and κ, respectively.

In view of ς<1, we conclude by Theorem 1 that our concerned example DUCNN has a unique equilibrium state (or fixed point), namely $x={\left(0,0,0\right)}^{⊤}$. In addition, by Theorem 2, it follows from the conclusion the LMIs (41) and (42) (with merely $\Psi_{2}$, $\Psi_{3}$ and Ω as the decision variables) are both feasible that $x={\left(0,0,0\right)}^{⊤}$ is almost surely exponentially stable. More precisely, combine (78) and (79) to arrive at

$$\begin{array}{cccc} \; & \underset{t\to +\infty }{lim sup}\frac{\ln\sum_{k=1}^{3}{\left|x_{k}\left(t\right)\right|}^{2}}{t}⩽-0.0947, & \; & M\mathrm{-a}.s., \end{array}$$

whenever $\sum_{k=1}^{3}{\left|x_{k}\left(t\right)\right|}^{2}>0$ for every $t\in R_{+}$, M-a.s. Let $x\left(t\right)={\left(x_{1}\left(t\right),x_{2}\left(t\right),x_{3}\left(t\right)\right)}^{⊤}$ be the (unique) state trajectory of our concerned example DUCNN satisfying the initial condition

(80) $$\left\{\begin{array}{ccc} x_{1}\left(t\right)=-5, & \; & dM\times dt\mathrm{-a}.e.\;\mathrm{in}\;\Gamma \times [-4,0],\\ x_{2}\left(t\right)=1, & \; & dM\times dt\mathrm{-a}.e.\;\mathrm{in}\;\Gamma \times [-4,0],\\ x_{3}\left(t\right)=3, & \; & dM\times dt\mathrm{-a}.e.\;\mathrm{in}\;\Gamma \times [-4,0]. \end{array}\right.$$

**Figure 1:**
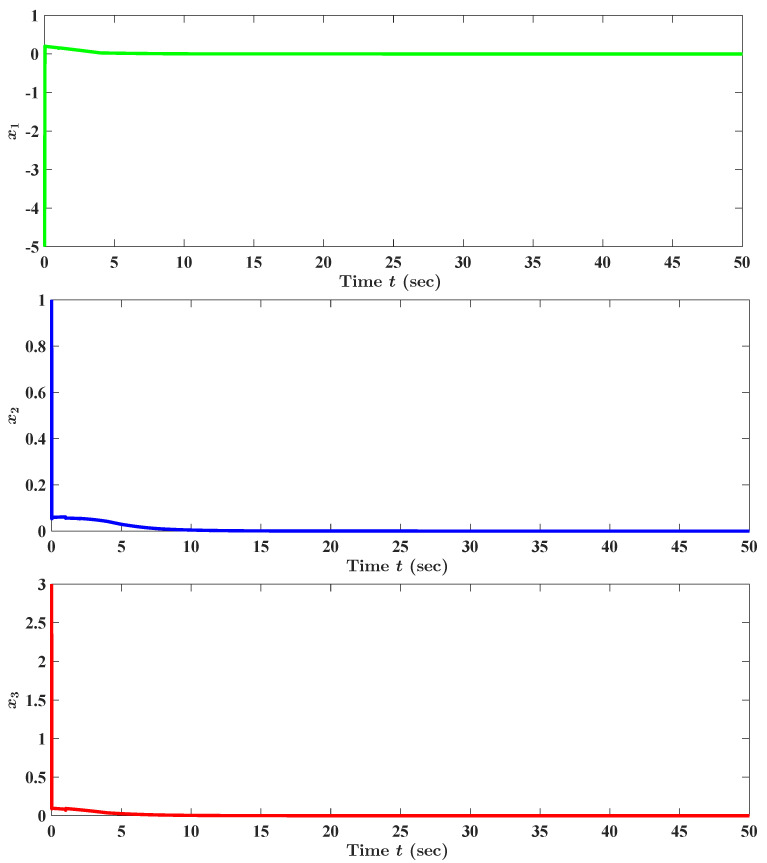
Numerical and graphical illustration of the criterion (see Theorem 1) ensuring the (unique) existence of equilibrium states (or fixed points) of our concerned DUCNNs (23), and the criterion (see Theorem 2) guaranteeing the almost surely exponential stability of the equilibrium states (or fixed points) of DUCNNs (23). $x\left(t\right)={\left(x_{1}\left(t\right),x_{2}\left(t\right),x_{3}\left(t\right)\right)}^{⊤}$, t∈[0,50], is the state trajectory of our concerned example DUCNN in this section (i.e., Section 4) fulfilling the initial condition (80).

By viewing Figure 1, we find readily that the state trajectory x(t) of our concerned example DUCNN supplemented by the initial condition (80) tends to 0, the equilibrium state (or fixed point) of the concerned example DUCNN, as time *t* escapes to infinity. To summarize, all the observations in this paragraph validate our theoretical results.

## 5. Concluding Remarks

We studied, in this paper, a class of DUCNNs, namely DUCNNs (23), driven by a one-dimensional canonical Liu process; see Section 2. Our concerned model DUCNNs include discrete time and finitely distributed time delay in transmission terms. In the context of uncertain dynamical systems, it seems to be new and difficult to investigate the influence of time delay on the long time behavior of state trajectories. Our research, in this paper, is inspired noticeably by the results in References [5,14,15,21,22,23,24,25,26,27,28,29,30,31,32,33,34], but we are faced with some new challenges. For example, it is not difficult to recognize that the Brownian motion is beneficial, in a certain sense, for proving almost surely the exponential convergence of state trajectories of stochastic NNs, while the canonical Liu process is actually ‘harmful’ for proving almost surely exponential convergence of state trajectories of uncertain NNs; see References [5,14,15,30,31,32,33,34]. Therefore, it seems to be much more challenging and laborious to perform convergence analysis on state trajectories for ‘indeterminate’ NNs driven by uncertain processes than for those driven by stochastic processes.

Based on some rudimentary analysis, we come up with a criterion (see (38)) under which our concerned model DUCNNs (23) were demonstrated, via a standard contraction mapping argument, to admit unique equilibrium states (or fixed points); see Theorem 1 and its proof for the details. By designing meticulously a class of Lyapunov–Krasovskii functionals, we brought forward, based on the analysis of our designed Lyapunov–Krasovskii functionals, a criterion (see (41) as well as (42)) to guarantee that the equilibrium states (or fixed points) of our concerned model DUCNNs (23) be almost surely exponentially stable; see Theorem 2 and its proof for the details. The aforementioned theoretical analysis and the corresponding results are collected in Section 3, and our theoretical results are ‘demonstrated’, numerically and graphically, to be actually effective.

Dynamical systems governed by CNNs of nonlinear differential equations driven by uncertain processes can be chaotic, in the sense some of the time series generated by (i.e., state trajectories of) the dynamical systems are of great complexity (for example, they are flexible and/or exhibit high entropy values). By exploiting machine learning, we can establish model to predict accurately flexible time series based on NNs. NNs whose state trajectories converging to their equilibrium states (or fixed points) perform better than those having divergent state trajectories. And therefore, our convergence criterion (see Theorem 2) helps us to design accurate CNN models to predict complex time series.

As pointed out in Section 1, to take sufficiently use of the after-effect in our concerned model DUCNNs (23), a class of Lyapunov–Krasovskii functionals, the main ingredients of this paper, were carefully created. Among the merits, general positive definite matrices are included in our designed Lyapunov–Krasovskii functionals to reduce the conservatism of our stability results. An interesting notion that is closely related to the main theme of our research in this paper is stabilization. By stabilization, we mean that extra control is added in uncertain NNs to guarantee that state trajectories of the controlled uncertain NNs converge to the equilibrium states (or fixed points). In the literature, various stabilization problems have been extensively studied for deterministic and stochastic NNs. Inspired by these observations, we shall work in the direction of designing suitable (impulsive control, intermittent control, quantized control, adaptive control, pinning control, sliding mode control, event-triggered control, and so forth) feedback control to stabilize DUCNNs.

As pointed out above, and by inspecting DUCNNs (23), it is not difficult to find that the model DUCNNs considered in this paper are driven by merely one dimensional canonical Liu processes. By reviewing all our mathematical derivations throughout this paper, it is not difficult to conclude that our methods can be adapted to treat similar problems associated to UCNNs (with or without time delay) driven by multi-dimensional Liu processes. Recently, the multi-dimensional Liu processes situation was considered in References [21,29]. Inspired by the results presented in these references, we plan to consider, in the near future, the problems concerning the existence and stability of equilibrium states (or fixed points) of DUCNNs driven by multi-dimensional Liu processes.

As can be seen above, we are merely focused, in this paper, on the existence and stability of equilibrium states (or fixed points). For NNs, equilibrium states (or fixed points) are special cases of periodic trajectories, and equilibrium states (or fixed points) as well as periodic trajectories latter are special cases of almost periodic trajectories. As mentioned in Section 1, in Reference [5], the problem concerning the stability of almost periodic trajectories of a certain class of NNs was considered. From the presentation of this reference, we can find that it is actually important to generalize the notion of equilibrium states (or fixed points) to that of (almost) periodic trajectories. In quite a few situations, NNs have no equilibrium state (or fixed point), but have (almost) periodic trajectories. In the procedure of investigating large time behavior of state trajectories of NNs, (almost) periodic trajectories act in nearly the same role as equilibrium states (or fixed points). We are therefore tempted to study the existence and stability of (almost) periodic trajectories of DUCNNs.

The notion of synchronizability is very close to that of stability. By synchronizability, we mean the phenomenon: Every difference trajectory of two NNs (the two NNs may have different structure) (i) tends to zero as time escapes to infinity or (ii) tends to zero as time approaches a finite instant (the so-called settling time), and remains to be zero constantly thereupon; see References [4,6,10,11]. As can be seen in many situations, complicated NNs display chaos phenomena. This would certainly bring about difficulty in the application of these NNs. To remove or attenuate the difficulty, synchronization control should be introduced into these chaotic NNs to improve their structural properties. Inspired by these brief disscusions here, we shall consider the (asymptotical, finite-time, fixed-time and/or pre-specified time) synchronization problem for DUCNNs.
